# Generation of Conformation‐Specific Monoclonal Antibodies for Integral Membrane Proteins

**DOI:** 10.1002/cpz1.70142

**Published:** 2025-05-26

**Authors:** Natalie Sheldon, Gunasekaran Dhandapani, Junhoe Kim, Cathy J. Spangler, Chengli Fang, Jumi Park, Prashant Rao, Eric Gouaux

**Affiliations:** ^1^ Vollum Institute Oregon Health & Science University Portland Oregon; ^2^ Howard Hughes Medical Institute Oregon Health & Science University Portland Oregon; ^3^ Present address: Calico Life Sciences LLC South San Francisco California; ^4^ These authors contributed equally to this work

**Keywords:** crystallization chaperones, fragment antigen‐binding region, glutamate receptors, proteoliposomes, single‐chain variable fragment

## Abstract

Antibodies and their antigen‐binding fragments, including fragment antigen‐binding domains (Fabs) and single‐chain variable fragments (scFvs), are extraordinary tools in all fields of biology, particularly in neuroscience, where they have been utilized for imaging, detection, and quantification studies. Most antibodies bind to unstructured or linear epitopes. Conformation‐specific antibodies, by contrast, bind to 3D epitopes, recognizing native conformations of the target antigen, and have proven highly useful in X‐ray crystallography as crystallization chaperones and in cryo‐electron microscopy as fiducial markers. Moreover, because conformation‐specific antibodies recognize 3D shapes of the antigen, they often have exquisite specificity and are useful in immunofluorescence studies and in isolation of antigen from native tissues. Over the past decade, our group has devoted effort to developing murine monoclonal antibodies (mAbs) against important synaptic receptors, particularly ionotropic glutamate receptors (iGluRs) and their auxiliary proteins. We have developed reproducible methods for generating high‐quality mAbs for structural, biochemical, and imaging studies. In this article, we show how to prepare proteoliposomes (PLs), carry out immunization and track the immune response, perform hybridoma generation, and analyze the specificity, cross‐reactivity, and competition of mAb binding via enzyme‐linked immunosorbent assay and fluorescence‐detection size‐exclusion chromatography. Our PL‐based method produces high‐affinity, conformation‐specific antibodies targeting diverse synaptic membrane receptors in 4 months. Here, we describe the relevant protocols in detail and document the mAbs, Fabs, and scFvs that we have produced against iGluRs and their auxiliary subunits. © 2025 The Author(s). Current Protocols published by Wiley Periodicals LLC.

**Basic Protocol 1**: Generation of conformation‐specific antibodies for integral membrane proteins

**Support Protocol 1**: Detection of conformational antibodies using ELISA

**Basic Protocol 2**: Expression and purification of monoclonal antibodies and their derivatives

**Support Protocol 2**: Concentration and clarification of insect cell supernatant for Fab purification

**Support Protocol 3**: Measurement of ionotropic glutamate receptor binding kinetics using Octet BLI System

## INTRODUCTION

Since the introduction of hybridoma technology (Pedrioli & Oxenius, 2021), production of recombinant monoclonal antibodies (mAbs) has been widely exploited in academia as well as in the biopharmaceutical industry (Kandari & Bhatnagar, 2023). Antibody‐antigen complexes are highly specific and represent one of the most robust noncovalent interactions occurring in nature. Antibodies have found extensive use in a broad range of applications, most notably in disease therapy, diagnostics, immunopurification, fluorescence studies, and biomarker detection due to their specificity and precise binding capabilities (Jin et al., 2022; Refaat et al., 2022). The structural modification of mAbs allows the generation of their derivatives, such as single‐chain variable fragments (scFvs) (Huston et al., 1988) and fragment antigen‐binding domains (Fabs) (Arlotta & Owen, 2019), and related constructs in combination with fluorescent molecules or small, electron‐dense gold nanoparticles (AuNPs) can further enhance the utility of antibodies and antibody fragments, enabling spectacular illumination of *in situ* antigen localization and dynamics (Matsui et al., 2024a; Wu et al., 2021).

One powerful utilization of antibody fragments has been in the realm of structure determination using X‐ray crystallography and single‐particle cryo‐electron microscopy (cryo‐EM). In the case of X‐ray crystallography, antibody fragments have facilitated the study of membrane proteins (MPs). For MPs, which are typically solubilized with detergents to form an MP‐detergent micelle complex, the dynamic and heterogeneous detergent micelle can hinder formation of protein‐protein interactions important for crystallization. Antibody fragments bound to the target MP can facilitate crystallization (Grisshammer, 2017; Warke & Momany, 2007) by enlarging the hydrophilic surface area of the MP and thereby providing additional surface area for formation of crystal contacts. In single‐particle cryo‐EM, antibody fragments are also powerful tools, especially for small complexes, because the addition of the scFv or Fab domain can act as a molecular fiducial, enlarging the size of the particle and facilitating particle alignment and image reconstruction (Bloch et al., 2021). However, a requirement for the use of antibody fragments for both X‐ray crystallography and single‐particle cryo‐EM is that the scFv or Fab must bind to a 3D epitope and thus be bound to the antigen via a conformationally stable interface. Antibody fragments that bind to linear, flexible epitopes are not useful because they are coupled via “floppy,” conformationally heterogenous interactions, forming complexes that are generally not useful for crystallization or for particle alignment.

Development of conformation‐specific mAbs has been challenging, however, because only limited portions of MPs are accessible for antibody binding, hampering the extent to which useful antibodies are produced. Moreover, MPs frequently populate multiple conformational states and possess flexible regions, thus conferring protein mobility, resulting in diminished success rates in antibody development. In addition, generation of high‐quality antibodies demands native, properly folded MPs, which in turn relies on robust and effective methods of MP preparation for antigen preparation and antibody screening.

Our lab has a track record of MP expression, purification, and structure determination, the latter of which often utilizes in‐house‐generated antibody fragments. Specific examples include the MP transporter ApcT, whose structure was elucidated in complex with the 7F11 Fab fragment, assisting molecular replacement (MR) using a known Fab structure as a search probe (Shaffer et al., 2009). Subsequently, the crystal structure of the glutamate‐gated chloride channel α (GluCl) protein in complex with 7G5 Fab demonstrated how an antibody fragment could boost the resolution of the crystals in comparison to the un‐complexed receptor (Hibbs & Gouaux, 2011). The bacterial analog of neurotransmitter sodium symporters, LeuT, showed how it was possible to raise conformationally specific antibodies to outward‐open and inward‐open conformations and to use the resulting complexes for structure determination of specific conformational states (Krishnamurthy & Gouaux, 2012). In addition, the *Drosophila* dopamine transporter (dDAT) was crystallized in the presence of the 9D5 Fab (Penmatsa et al., 2013, 2015; Wang et al., 2015), highlighting the utility of antibody fragments in crystallization of challenging eukaryotic MPs. Our studies, along with those from other laboratories, have demonstrated the successful use of antibody fragments in X‐ray crystallography, single‐particle cryo‐EM, and cryo‐electron tomography (cryo‐ET) studies involving a diverse range of MPs, including G‐protein‐coupled receptors (GPCRs) (Choe et al., 2011; Maeda et al., 2018; Robertson et al., 2022), bacterial and toxin‐related proteins (Nguyen & Song, 2021; Rathnayake et al., 2024), viral glycoproteins (Chen et al., 2022; Gui et al., 2017) and neurotransmitter receptors and ion channels (Matsui et al., 2024; Selvakumar et al., 2022; Wang et al., 2023). These findings highlight the great utility of antibody fragments in molecular and cellular structural biology.

MPs that play a particularly important role in human physiology and medicine are located at the chemical synapses of the brain, the communication junctions between nerve cells. In the vertebrate brain, ionotropic glutamate receptors (iGluRs) mediate the majority of excitatory signaling and participate in a variety of cognitive and neural developmental processes, including memory, learning, motor coordination, and synaptic plasticity (Traynelis et al., 2010). iGluRs fall into three major classes: α‐amino‐3‐hydroxy‐5‐methyl‐4‐isoxazolepropionic acid receptor (AMPAR), *N*‐methyl‐D‐aspartate receptor (NMDAR), and kainate receptors. The architecture and spatial arrangement of iGluRs and their auxiliary proteins exhibit profound specificity and differences across various brain regions (Traynelis et al., 2010). The heterotetrameric NMDARs are typically composed of three different subunits: GluN1, GluN2 (GluN2A‐D), and GluN3 (GluN3A‐B). Tetrameric NMDARs have two obligatory GluN1 and two GluN2 and/or GluN3 subunits to form a functional receptor (Chou et al., 2020; Stroebel & Paoletti, 2021). AMPARs are heterotetrameric assemblies comprising four different subunits: GluA1‐A4 (Gouaux, 2004). The GluA2 subunit, present in most AMPARs, gives rise to receptors with reduced calcium permeability. Furthermore, various auxiliary subunits, such as the transmembrane AMPAR regulatory proteins (TARPs), cornichon homologs 2 and 3 (CNIH2, 3), and GSG1L protein, control basic characteristics of AMPAR gating, channel conductance, and pharmacology.

Subunit‐specific antibodies targeting NMDARs and AMPARs are essential for investigating receptor spatial distribution and function in the brain. For example, anti‐GluA1 and anti‐GluA2 antibodies, such as 11B8 and 15F1, have been used in universal Point Accumulation for Imaging in Nanoscale Topography (uPAINT) to study the dynamics of glutamatergic AMPARs on the plasma membrane of live hippocampal neurons under physiological conditions (Giannone et al., 2010; Youn et al., 2023). The development of subunit‐specific anti‐AMPAR antibodies has enabled the analysis of AMPAR composition, dynamics, and nanoscale organization within the hippocampus using single‐molecule total internal reflection fluorescence (TIRF) microscopy (Yu et al., 2021), and these antibodies also have broader applications in immunofluorescence studies for precise receptor localization (Carolin et al., 2019; Hosokawa et al., 2021). The development of specific, high‐affinity iGluR‐specific antibodies enables native receptor purification, stoichiometric analysis, subunit mapping, receptor localization, and high‐resolution structural characterization using electron microscopy techniques (Bissen et al., 2019).

We present a set of robust and reproducible protocols for developing antibodies against iGluRs and their auxiliary subunits using the proteoliposome (PL) immunization method. This approach is routinely used in our laboratory for generating mAbs and antibody fragments against a broad range of antigens for structural, biochemical, and imaging studies. To provide a comprehensive methodology, we outline PL preparation, immunization, antibody generation, screening, and evaluation in Basic Protocol 1, and the expression and purification of cognate mAbs and Fabs are detailed in Basic Protocol 2. To support these methods, we include three support protocols: Support Protocol 1 describes an enzyme‐linked immunosorbent assay (ELISA) for screening serum and hybridoma supernatants to assess antigen specificity, cross‐reactivity, and conformation specificity of antibodies; Support Protocol 2 describes the processing of insect cell culture supernatant for affinity purification; and Support Protocol 3 provides a detailed procedure for biolayer interferometry (BLI) to evaluate high‐affinity antibodies.

*NOTE*: Approval from the institutional ethics review committee must be obtained for animal‐related work. In our case, all experiments were conducted in compliance with the Oregon Health & Science University Institutional Animal Care and Use Committee (IACUC).

## Basic Protocol 1 GENERATION OF CONFORMATION‐SPECIFIC ANTIBODIES FOR INTEGRAL MEMBRANE PROTEINS

**Figure 1:**
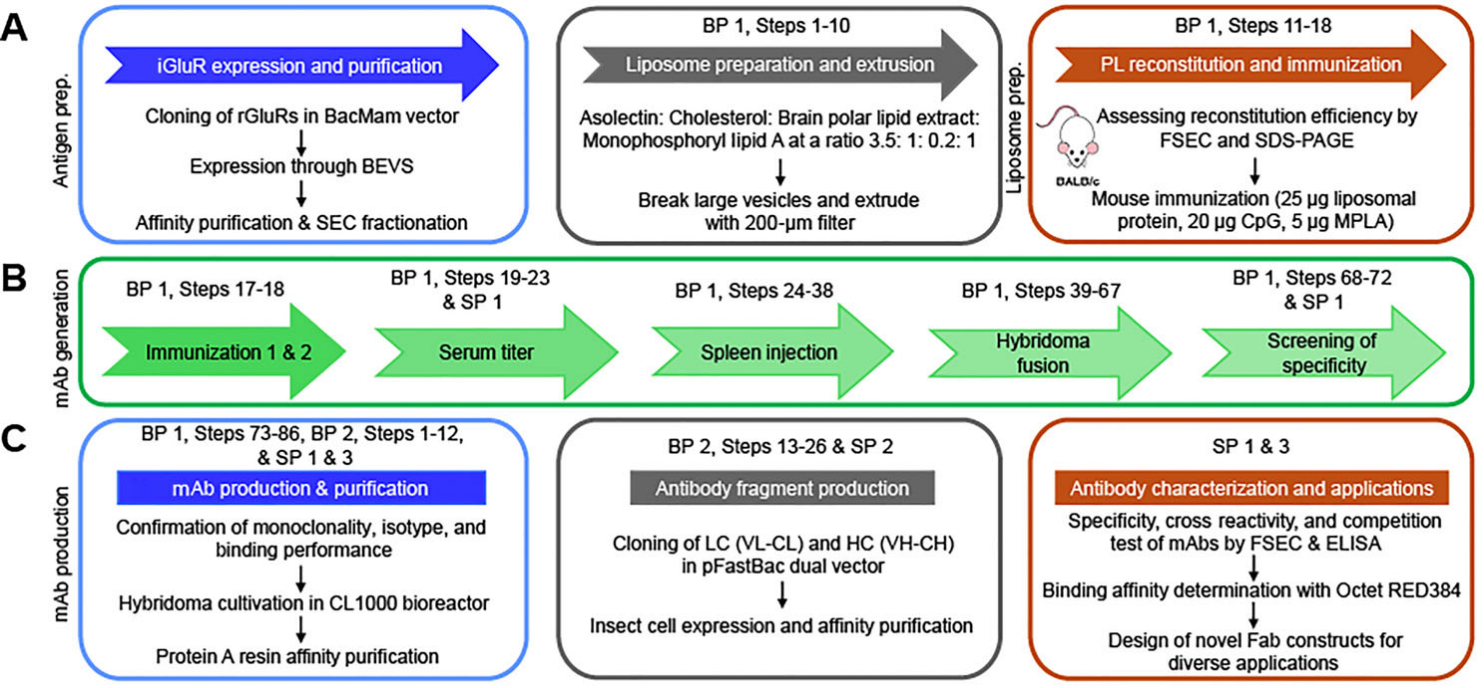
Timeline of mouse immunization, mAb generation, and hybridoma screening. (**A**) Overview of the procedures for iGluR expression, large‐scale mAb production, liposome and PL preparation, and associated confirmation methods, outlined in BP 1, steps 1 to 16. (**B**) Six‐ to eight‐week‐old BALB/c mice were immunized intraperitoneally with 100 µl of 25 µg liposomal antigen, 20 µg CpG, and 5 µg MPLA, administered twice with a 2‐week interval, following IACUC guidelines (BP 1, steps 17 and 18). The procedures for blood serum titration, spleen injection, hybridoma fusion using the classical PEG method, and single‐cell hybridoma isolation are detailed in BP 1, steps 19 to 86. (**C**) Hybridoma cultivation, mAb production, and generation of novel antibody derivative constructs and their purification and characterization methods are described in BP 2, SP 1, SP 2, and SP 3. Abbreviations: BP 1 = Basic Protocol 1, BP 2 = Basic Protocol 2, SP 1 = Support Protocol 1, SP 2 = Support Protocol 2, SP 3 = Support Protocol 3, BEVS = baculovirus expression vector system.

**Figure 2:**
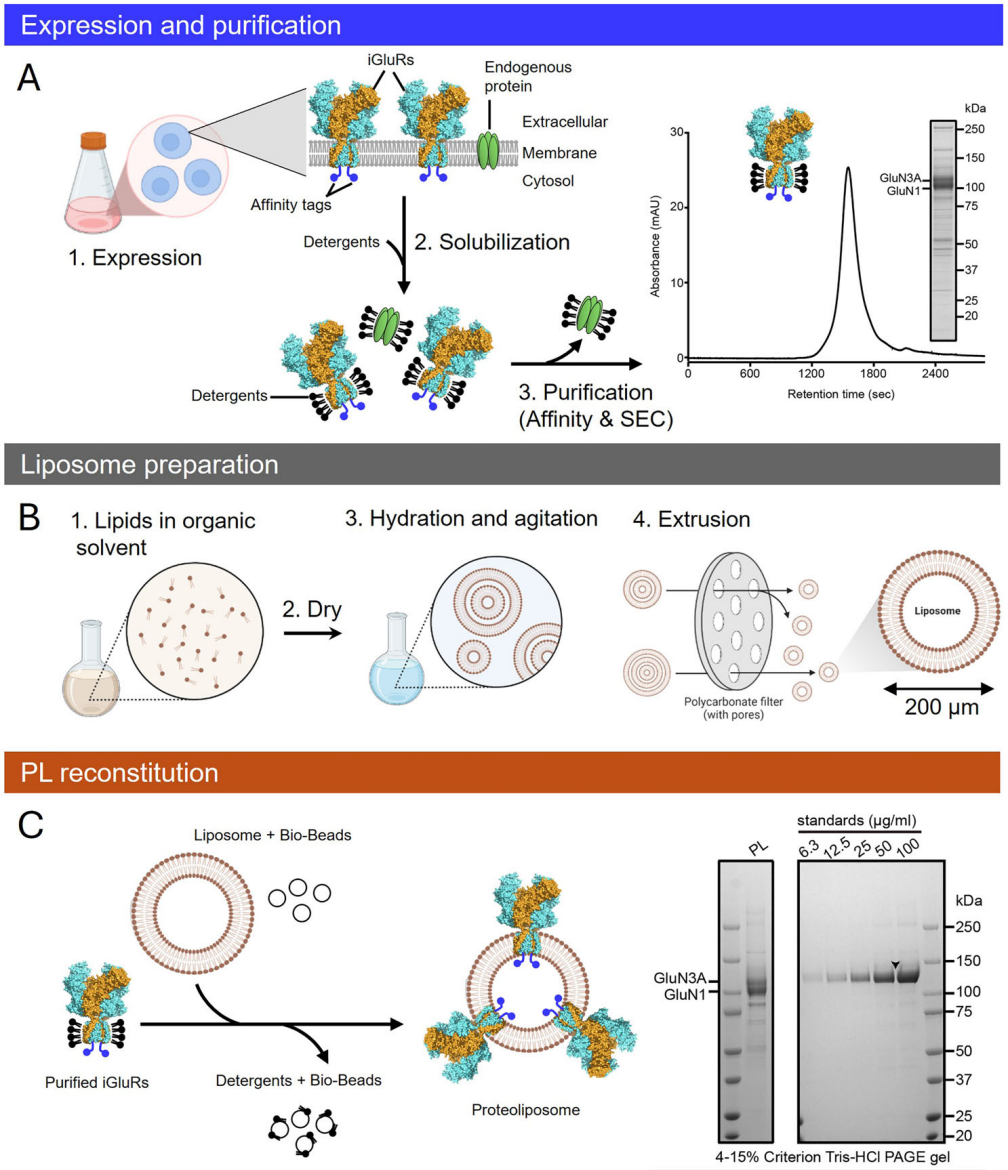
iGluR expression, purification, and liposome reconstitution. (**A**) Schematic overview of iGluR expression and purification in a heterologous system. Functional iGluRs embedded in the cellular membrane are solubilized with detergents and purified using affinity tags (blue dots), followed by SEC. The quality of the purified iGluR protein is assessed by FSEC (Ex280/Em315 nm) and SDS‐PAGE. (**B**) Schematic representation of the liposome preparation process. (**C**) PL reconstitution is carried out by combining unilamellar liposomes [prepared as in (B)] with purified iGluRs [from (A)] and bio‐beads to remove detergent, facilitating iGluR incorporation into liposomes. Representative SDS‐PAGE gels and quantification of PL reconstitution are shown. Certain illustrations in the "Expression and purification" and "Liposome preparation" sections were adapted in BioRender.

We present a detailed protocol covering antigen and liposome preparation, PL reconstitution, immunization schedules, hybridoma fusion, screening methods with immune serum and hybridoma supernatant, and the production of subunit‐specific antibody fragments for structural and biochemical studies (Fig. 1). Candidate iGluR genes were cloned in the pEG BacMam vector and expressed in HEK293S GnTI− cells/tSA201 cells through baculovirus transduction using previously established methods (Goehring et al., 2014) (Fig. 2A).

### Materials

Acetone (Sigma, cat. no. 179124)
Deionized water
Chloroform (Sigma, cat. no 650471)
Asolectin (Sigma, cat. no. 11145)
Cholesterol (Sigma, cat. no. C8667)
Brain polar lipid extract (Avanti Polar Lipids, cat. no. 141101)
Monophosphoryl lipid A (MPLA; Avanti Polar Lipids, cat. no. 699800)
Tris‐buffered saline (TBS; see recipe)
Liquid nitrogen
Protein solution (containing iGluR antigen)
Solubilization detergent [lauryl maltose neopentyl glycol (LMNG), Anatrace, cat. no. NG310, or n‐dodecyl‐β‐d‐maltopyranoside (DDM), Anatrace, cat. no. D310]
2 mg/ml CpG oligonucleotide (see recipe)
1 mg/ml MPLA (see recipe)
PBS (Corning/Cellgro, cat. no. 21040CV)
BALB/c mice (Charles River, cat. no. 028)
Isoflurane (Sigma, cat. no. 792632)
Buprenorphine extended release (Wedgewood Pharmacy)
Puralube veterinary ophthalmic ointment (Covetrus, cat. no. 008897)
ChloraPrep (BD, cat. no. 930480)
Frozen myeloma cells (P3 × 63Ag8.653 myeloma cells, ATCC, cat. no. CRL‐1580)
Myeloma growth medium (see recipe), 37°C
RPMI medium without fetal bovine serum (FBS; ATCC, cat. no. 30‐2001), 37°C
70% (v/v) ethanol (Sigma, cat. no. 277649)
Red Blood Cell Lysing Buffer (Millipore Sigma, cat. no. R7757)
Trypan blue (Corning/Cellgro, cat. no. 25‐900‐CL)
50% (w/v) polyethylene glycol (PEG; PEG 1450, Millipore Sigma, cat. no. P7181)
HAT selection medium (see recipe; make fresh), 37°C
ELISA protein denature solution (see recipe; make fresh)
TBS (see recipe) with 0.2% (v/v) Tween 20 (Fisher Scientific, cat. no. BP337) and 0.2% (w/v) bovine serum albumin (BSA)
Initial hybridoma growth medium (see recipe), 37°C
Opti‐MEM (Gibco, cat. no. 31985070) with 10% (v/v) ultra‐low‐IgG FBS (Thermo Fisher Scientific, cat. no. 16250078) and 20% (v/v) sterile dimethyl sulfoxide (DMSO; Millipore Sigma, cat. no. D2650)

Mouse isotyping kit (Bio‐Rad, cat. no. MMT1)
100‐ml glass round‐bottom flask (Fisher, cat. no. 100672B)
Rotary evaporator with 35°C water bath (Buchi, cat. no. R100)
37°C and 40°C water baths (Fisher Scientific Isotemp 210)
Liquid hazardous waste container
Vacuum desiccator (Corning, cat. no. 3121‐150) with desiccant
Vortex
Bath sonicator (Branson B200)
Extruder (LIPEX thermobarrel extruder, Evonik, cat. no. TF.001, or Mini‐Extruder, Avanti Polar Lipids, cat. no. 610000)
0.2‐ and 0.4‐µm Whatman nucleopore track etch membrane filters Whatman nucleopore track etch membranes 0.2 µm, 25 mm circles, Cytiva, cat. no. 10417006, and Whatman nucleopore track etch membranes 0.4 µm, 25 mm circles, Cytiva, cat. no. 10417106)
Drain disc (Cytiva, cat. no. 230600)
Compressed nitrogen gas cylinder
50‐cml onical tubes (Fisher, cat. no. 0553913)
Optima MAX‐XP tabletop ultracentrifuge (Beckman, cat. no. 393315)
Bio‐beads (Bio‐Beads SM‐2 Adsorbent, Bio‐Rad, cat. no. 1523920)
Rotator
300‐µl insulin syringes with 29G needles (Covetrus, cat. no. 037322)
20G needles (BD, cat. no. 305176)
Capillary blood collection tubes (Sarstedt, cat. no. 20.1282.100)
Microcentrifuge (Eppendorf, cat. no. 5406000240)
TL100 ultracentrifuge
Portable anesthesia machine
Clippers (Braintree Scientific, cat. no. CLP‐41590)
Heated surgery stage (Braintree Scientific, cat. no. 39OP)
Small toothed forceps (Fine Science Tools, cat. no. 11053‐10)
Fine iris scissors (Fine Science Tools, cat. no. 14094‐11)
Curved forceps (Fine Science Tools, cat. no. 11041‐08)
5‐0 Biosyn CVF‐21 (Medline, cat. no. USUSM432)
Needle holder (Fine Science Tools, cat. no. 12500‐12)
Sorvall Legend XFR centrifuge (Thermo Fisher Scientific, cat. no. 75004539)
T25, T75, and T175 flasks (cell culture flask 25 cm^2^, Thermo Scientific, cat. no. 130189; cell culture flask 75 cm^2^, Thermo Scientific, cat. no. 130190; and cell culture flask 175 cm^2^, Thermo Scientific, cat. no. 130191)
Paper towels
Tissue culture hood (Thermo Fisher 1300 Series A2)
Forceps and scissors, sterile
10‐cm tissue culture dishes (Falcon, cat. no. 353003)
20G needles
Cell dissociation mesh screen, sterile (Chemglass Life Sciences, cat. no. CLS‐5020)
Hemacytometer (Hausser Scientific, cat. no. 1492)
Inverted microscope (Nikon Eclipse TS100)
10‐ml serological pipets
96‐well tissue culture plates (Fisher, cat. no. FB012931)
Disposable reagent reservoirs, sterile (100 ml, VWR, cat. no. 89094‐658)
12‐channel pipettor, sterile
96‐well transfer plates (Thermo Scientific, cat. no. 269620)
70°C heat block (VWR, cat. no. 76549‐936)
24‐well tissue culture plates (Falcon, cat. no. 353047)
2‐ml cryogenic vials (Corning, cat. no. 430488)
Mr. Frosty freezing container (Thermo Fisher Scientific, cat. no. 51000001)
Liquid nitrogen storage dewar
Additional reagents and equipment for fluorescence‐detection size‐exclusion chromatography (FSEC) (Kawate & Gouaux, 2006), SDS‐PAGE (see Current Protocols article: Gallagher, 2012), ELISA (see Support Protocol 1), and mouse euthanasia

*NOTE*: All solutions and equipment coming into contact with cells must be sterile, and proper sterile technique should be used accordingly.

*NOTE*: All culture incubations are performed in a 37°C, 5% CO_2_ incubator (Thermo Forma 3110) unless otherwise specified.

### Day 1: Liposome preparation

1 Wash a clean 100‐ml glass round‐bottom flask with 5 ml acetone, then deionized water, and then 5 ml chloroform.
2 Dissolve asolectin/cholesterol/brain polar lipid extract/MPLA at a ratio of 3.5:1:0.2:1 in 10 ml chloroform in the 100‐ml glass round‐bottom flask.
  Always use glassware when handling lipids to prevent adsorption and ensure chemical compatibility.
3 Connect the flask to a rotary evaporator with a 35°C water bath. Submerge the flask in the water bath up to the neck. Turn the speed to 120 rpm and evaporate the chloroform under vacuum in the water bath for ≥1 hr so that the lipids form a thin film around the flask. Discard the removed chloroform in an appropriate liquid hazardous waste container.
4 Place the flask in a vacuum desiccator with desiccant and apply vacuum. Allow the lipids to dry overnight under vacuum to completely remove the chloroform.

### Day 2: Liposome resuspension and extrusion (Fig. 2B)

5 Add 10 ml TBS to the dried lipid film and mix thoroughly by vigorous pipetting or vortexing until a homogeneous lipid suspension is achieved.
6 Flash‐freeze the lipid mixture by immersing the flask in liquid nitrogen and rotating it to form a thin layer of the lipid mixture around the flask. Then, rapidly thaw the lipid mixture by placing the flask in a 40°C water bath. Place the flask into a bath sonicator and sonicate for 1 min. Repeat the freeze, thaw, and sonicate cycle at least 10 times, up to 15 times, until the mixture becomes a milky suspension.
  Freeze‐thaw cycles are crucial for breaking down larger liposomes. We recommend repeating step 6 until a homogeneous solution is achieved. Inadequate lipid solubilization can negatively impact both the yield and the quality of the liposomes.
7 Extrude the liposomes through a 0.4‐µm Whatman nucleopore track etch membrane filter supported by a drain disc, applying pressure with a compressed nitrogen gas cylinder and adding 1 ml lipids to the chamber at a time. Collect the extruded liposomes in a 50‐ml conical tube kept on ice. Replace the filter when the extrusion pressure reaches 500 psi.
8 Use a 0.2‐µm Whatman nucleopore track etch membrane filter to extrude the lipids following the same procedure as in step 7.
9 Next, use two 0.2‐µm Whatman nucleopore track etch membrane filters stacked together to extrude the lipids following the same procedure as in step 7. Extrude the lipids a minimum 10 times.
  The mixture should appear translucent. As you continue, you can increase the sample volume in the chamber, and the extrusion process will become easier.
  Adding too much lipid mixture into the extruder at once can clog the filter and result in sample loss.
10 Spin the lipids for 45 min at 100,000 × *g* in an Optima MAX‐XP tabletop ultracentrifuge.
  Pellets can be stored at –80°C for months before reconstituting with protein.

### Day 3: Antigen reconstitution into liposomes (Fig. 2C)

11 Resuspend the lipid pellet in protein solution.
  For good antigenic properties, a protein/lipid (w/w) ratio of 1:50 to 1:100 is recommended. The lower the volume of protein, the better incorporation into liposomes.
12 To interrupt the liposomes, incubate the protein/liposome solution with the appropriate solubilization detergent (LMNG or DDM) for 30 min on ice.
  A final detergent concentration recommendation for LMNG is 1 mM and for DDM is 3 mM.
  The same detergent concentration is applied to all proteins used as antigens, although it may sometimes require screening with different classes of MPs.
13 Remove excess detergent from the PLs by incubating with 200 to 250 mg prepared bio‐beads per 1 ml PLs containing ∼500 µg protein. Incubate at 4°C with rotation. Repeat the incubation process three times, with fresh bio‐beads for each incubation: twice for 2 hr each and once overnight.
  It is recommended to wash Bio‐Beads SM‐2 Adsorbent in methanol prior to use. Incubate the bio‐beads in methanol for 30 min with rotation and then centrifuge 5 min at 1000 × g. Discard the methanol supernatant and wash the beads thoroughly with PBS or the desired buffer to remove any residual methanol before further use.

### Day 4: Analysis of antigen incorporation efficiency

14 To check for protein incorporation into liposomes, solubilize 5 µl in 50 to 100 µl detergent and perform FSEC (Kawate & Gouaux, 2006).
  For FSEC, the analyte was centrifuged 20 min at 86,500 × g, and a fraction of the supernatant was loaded onto a pre‐equilibrated Superose 6 Increase 10/300 GL column (Cytiva, cat. no. 29091596) with FSEC buffer (see recipe), running at a flow rate of 0.5 ml/min. For the GluN1/N3A NMDAR, 20 nM GFP‐fused receptor protein was loaded onto the column using FSEC buffer (see recipe). The eluent from the SEC column was passed through a fluorometer, with excitation at 480 nm and emission at 510 nm for GFP fluorescence and excitation at 280 nm and emission at 315 nm for tryptophan fluorescence. Calibration with known quantities of GFP has shown that 1 to 10 ng GFP can be readily detected (Hattori et al., 2012; Kawate & Gouaux, 2006).
15 Take another 5 µl of solubilized PLs and load this mixture on an SDS‐PAGE gel (see Current Protocols article: Gallagher, 2012) along with known amounts of protein to estimate incorporation efficiency (Fig. 2C).
16 Centrifuge PLs for 45 min at 100,000 × *g* in an Optima MAX‐XP tabletop ultracentrifuge.
  PLs can be aliquoted and frozen at –80°C for months, followed by mild thawing on ice for use.

### Days 5 and 19: Immunizations 1 and 2

**Figure 3:**
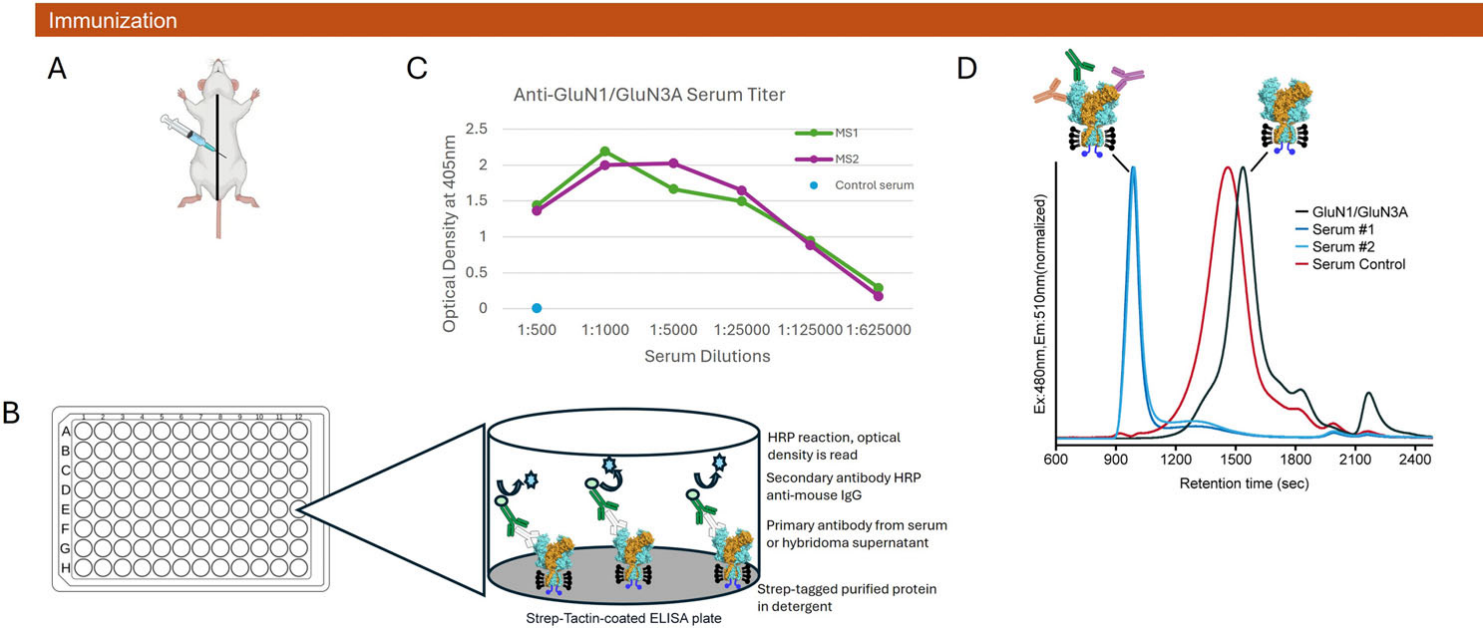
Immunization and antibody screening in GluN1/GluN3A NMDAR mice. (**A**) Intraperitoneal injection is performed just off the midline at the hip level on the left side of the mouse. (**B**) ELISA wells coated with Strep‐Tactin capture Strep‐tagged iGluR protein in its native, folded conformation. Primary antibodies bind to the iGluR, and binding is detected using an HRP‐conjugated anti‐mouse IgG secondary antibody. (**C**) Serum ELISA for immunized GluN1/GluN3A NMDAR mice shows immune reactivity against the target, with titers exceeding 1:125,000, whereas no titer is detected in control serum. (**D**) FSEC analysis of PL‐incorporated GluN1/GluN3A receptors with sera from two immunized mice and a non‐immunized mouse control after two rounds of immunizations. FSEC traces (Ex480/Em510 nm) show the shifts of the GFP‐conjugated GluN1/GluN3A receptor peak, indicating the binding of antibodies.

17 Mix 25 µg liposomal protein, 20 µg CpG oligonucleotide (from 2 mg/ml), 5 µg MPLA (from 1 mg/ml), and PBS to 100 µl total volume per BALB/c mouse to be immunized. Draw up each immunization into a 300‐µl insulin syringe with a 29G needle.
18 Deliver the immunization by intraperitoneal injection 3 to 5 mm lateral to the midline and just above hip level on the mouse's left side (Fig. 3A) on days 5 and 19.

### Day 31: Blood draw and serum titer

19 Puncture the medial saphenous vein with a 20G needle and collect blood by capillary into a capillary blood collection tube. Centrifuge the blood for 10 min at 2000 × *g* in a microcentrifuge and carefully pipet the clear serum into a separate tube. Store at 4°C for immediate use.
20 Screen immune serum for target specificity via ELISA as described in Support Protocol 1 (Fig. 3B and 3C).

### Day 31: Screening serum for protein shift by FSEC

21 Mix 50 nM GFP/protein of interest with 1, 3, or 5 µl serum sample from immunized mice or controls. Incubate the samples for 30 min on ice.
22 Centrifuge samples for 40 min at 70,000 × *g*, 4°C, in a TL100 ultracentrifuge.
23 Move the supernatant to a new tube and analyze 50 µl by FSEC (see step 14). Allow 1 hr for each sample to be analyzed by FSEC. Store samples at 4°C until analysis.
  Mice with the best serum response should display a shift of the protein peak toward the void, indicating antibodies are bound to the conformationally intact protein (Fig. 3D).
  The results of the serum analysis by FSEC and ELISA should be used to determine which mice should be used for the fusion or if a third immunization should be given.

### Day 51: Spleen injections

24 Mix 10 µg liposomal protein with PBS to 50 µl total per mouse and draw it up into a 300‐µl insulin syringe with 29G needle.
  The procedure detailed in steps 24 to 38 is performed under sterile conditions by trained personnel following all IACUC guidelines.
25 Anesthetize a mouse with metered 5% isoflurane with a 100% oxygen carrier for induction and maintain anesthesia at metered 1% to 3% isoflurane with a 100% oxygen carrier during surgery.
26 Clip the fur on the left side of the mouse from the rib cage to the flank and secure the mouse in right lateral recumbency on a heated surgery stage.
27 Inject the mouse with 0.5 to 1 mg/kg buprenorphine extended release subcutaneously over the shoulder.
28 Ensure proper depth of anesthesia by checking for absence of toe pinch reflex and counting respiration rate.
29 Apply Puralube veterinary ophthalmic ointment to the eyes of the mouse.
30 Apply ChloraPrep to the skin of the clipped side of the mouse.
31 Using small toothed forceps, grasp and tent the skin over the top of the spleen (which should be visible through the skin), 3 to 4 mm from the ventral end of it. Using fine iris scissors, cut the tented skin perpendicular to the skin and the mouse's body so that when the skin is released, the incision is straight. Use the tip of the iris scissors to gently stretch the incision lengthwise.
32 Grasp the fascia and muscle layer directly beneath the skin incision with the toothed forceps and cut through with the iris scissors in similar fashion to the skin incision (see step 31). If necessary, extend the incision either by cutting farther with the iris scissors or by bluntly spreading the incision with the tip of the scissors.
  Unless the spleen is scarred and adhered to the body wall (common), it should fall away, and you will just see a dead space or hole where you made your cut; this is the abdominal cavity.
33 Gently grasp the fatty pedicle on the underside of the spleen with the curved forceps and draw the spleen out of the body cavity. If the spleen is adhered to the body wall, do not try to dissect it away from the body wall; simply exteriorize as much as possible, extending the incision if necessary.
  The spleen is fragile and easy to damage, so do not grasp it directly with instruments.
34 Grasping the splenic fatty pedicle for countertraction, gently advance the needle tip (see step 24) into the spleen, bevel up, along the length of the spleen without exiting, approximately 3 to 5 mm, as far as possible while still being able to visualize the needle in the spleen. Then, slowly begin the injection while also slowly withdrawing the needle. Once all the antigen has been injected, pause momentarily with the needle tip still in the spleen, giving the antigen a little time to disperse and minimizing any leakage from the injection site. If there is any bleeding from the splenic injection site, apply gentle pressure until it stops.
35 Return the spleen to the abdominal cavity.
36 Close the abdominal wall with a cruciate stitch of 5‐0 Biosyn CVF‐21 using a needle holder.
37 Close the skin with simple interrupted 5‐0 Biosyn, typically 2 or 3 stitches.
38 Remove the anesthesia and allow the mouse to recover completely.

### Days 46 to 52: Myeloma cell growth

Cell cultures are a potential biohazard. Work in an approved laminar flow hood using aseptic techniques. Check institutional and governmental guidelines for recommended protective clothing and proper disposal of waste before performing experiments.

39 One week before the fusion, quick‐thaw a vial of frozen myeloma cells in a 37°C water bath until a small ice chip remains, ∼2 min.
40 Add cells to 10 ml pre‐warmed myeloma growth medium in a 50‐ml conical tube. Centrifuge 10 min at 130 × *g* in a Sorvall Legend XFR centrifuge. Remove medium and resuspend cells in 50 ml fresh myeloma growth medium and then transfer to a T75 flask. Grow in an incubator with 37°C, 5% CO_2_.
41 Four days before the fusion, collect myeloma cells and centrifuge 10 min at 130 × *g*. Remove medium and plate the cells in 200 ml myeloma growth medium in a T175 flask.
42 Two days before the fusion, collect myeloma cells and centrifuge 10 min at 130 × *g*. Remove medium and plate the cells in 400 ml fresh myeloma growth medium in two T175 flasks.
43 The day before the fusion, collect myeloma cells and centrifuge 10 min at 130 × *g*. Remove medium and plate the cells in 600 ml fresh myeloma growth medium in three T175 flasks.

### Day 53: Hybridoma fusion with polyethylene glycol

44 Collect the myeloma cells by centrifugation for 10 min at 130 × *g*.
45 Discard the supernatant and resuspend the cell pellet in a total of 90 ml RPMI medium without FBS.
  It is important to thoroughly wash myeloma and spleen cells in serum‐free medium before the fusion; residual serum will inhibit PEG fusion.
46 Euthanize the mice. Soak a paper towel in 70% (v/v) ethanol and place in the work area of the tissue culture hood. Soak a mouse with 70% (v/v) ethanol and place it on the paper towel in the hood.
  Cell culture, spleen isolation, and dissociation steps should be conducted under sterile conditions to prevent contamination.
47 Using sterile forceps and scissors, carefully remove any fur and cut through the skin on the left side to expose the chest cavity without cutting through the peritoneal layer.
48 Using new sterile forceps and scissors, cut through the peritoneal layer to expose the abdominal cavity. Carefully remove the spleen and place on the lid of a 10‐cm tissue culture dish, inverted to hold 10 ml RPMI medium without FBS.
49 Partially remove a 20G needle from the sterile cap and bend it 90°. Holding the spleen with a pipet tip on one half, poke holes in the opposite half using the bent needle. Then, using the bent edge like a hockey stick and a sterile cell dissociation mesh screen, gently but firmly press the red cells out of the spleen until only the casing remains. Repeat on the other half of the spleen. Remove and discard the casing.
  Using a sterile mesh screen helps remove the spleen casing from the cells.
50 Transfer the 10 ml medium and spleen cells to a 50‐ml conical tube. Wash the dish three times with 10 ml RPMI medium without FBS, collecting each wash in the 50‐ml tube. Fill the tube up to 45 ml total.
51 Centrifuge myeloma cells (step 45) and spleen cells (step 50) for 10 min at 130 × *g*.
52 Remove medium from the spleen cells and add 10 ml Red Blood Cell Lysing Buffer.
53 While red blood cells are lysing, remove medium from myeloma cells and gently resuspend them in 45 ml RPMI medium without FBS.
54 Add 35 ml RPMI medium without FBS to the spleen cells in Red Blood Cell Lysing Buffer and centrifuge the spleen/myeloma cells for 10 min at 130 × *g*.
55 Carefully remove medium and gently resuspend the spleen cells in 20 ml RPMI medium without FBS. Then, fill the tube up to 45 ml. Centrifuge spleen cells for 10 min at 130 × *g*.
56 While spleen cells are being centrifuged, remove medium from myeloma cells and gently resuspend them in 20 ml RPMI medium without FBS. Count cells using trypan blue exclusion using a hemacytometer and inverted microscope.
57 Remove medium from spleen cells and gently resuspend them in 20 ml RPMI medium without FBS. Count cells by trypan blue exclusion.
58 To the 20 ml spleen cells, add sufficient volume of myeloma cells to give a ratio of 2 spleen cells to 1 myeloma cell.
  Typically, this is 60 million spleen cells to 30 million myeloma cells for one spleen.
  All spleen cells are used for fusion.
59 Centrifuge the combined cells for 10 min at 130 × *g*.
60 Remove the medium from the pellet and add 1 ml pre‐warmed PEG dropwise over 1 min while gently tapping the tube to help break up the pellet.
61 Gently tap the tube for an additional 1 min to help promote cell fusion.
62 Add 1 ml RPMI medium without FBS dropwise over 1 min.
63 Add 7 ml RPMI medium without FBS dropwise from a 10‐ml serological pipet over 3 min.
64 Centrifuge the cells for 10 min at 50 × *g* to collect the fused cells.
65 Remove the medium from the cells. Gently resuspend the pellet in 10 ml HAT selection medium and then bring the total volume to 225 ml per spleen.
66 Add 300 µl per well to 96‐well tissue culture plates using a sterile disposable reagent reservoir and a sterile 12‐channel pipettor.
67 Incubate fusion plates at 37°C, 5% CO_2_ for 12 days without disturbing.

### Day 66: Initial fusion screening ELISA

68 In the tissue culture hood, aseptically transfer 50 µl hybridoma supernatant medium from the fusion plates to 100 µl TBS in 96‐well transfer plates for a 1:3 dilution.
  Be sure that the fusion plates and the dilution plates are labeled correspondingly.
69 Perform an ELISA following Support Protocol 1, replacing the serum dilutions with hybridoma supernatants diluted to 1:30.

### Day 66: Native and denaturing ELISA

**Figure 4:**
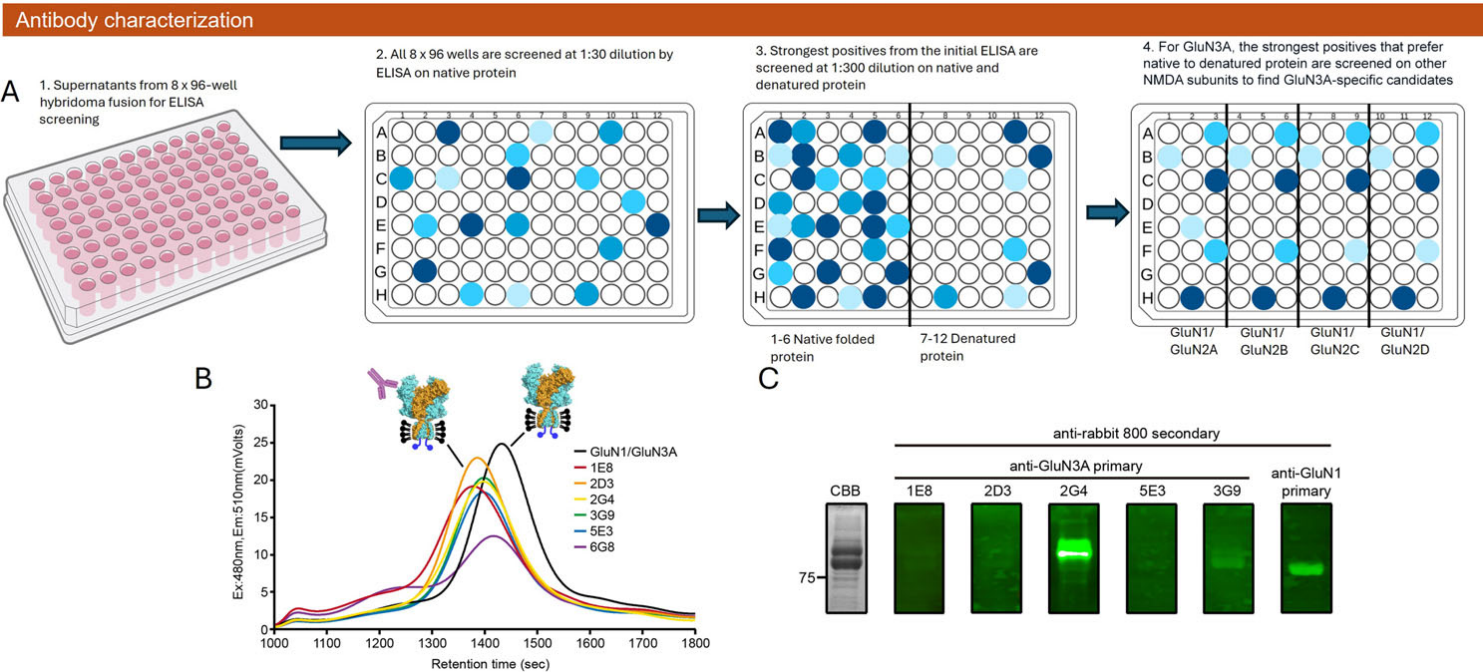
Screening and characterization of anti‐GluN3A NMDAR antibody candidates. (**A**) Hybridoma fusion using spleen cells, followed by plating into eight 96‐well plates and culturing for 12 days. Supernatants are screened by ELISA at a 1:30 dilution to detect antibody binding to native protein. Wells with strong signals are retested at a 1:300 dilution with both native and denatured protein. Antibodies that preferentially bind native protein are considered potential 3D epitope binders, and those that prefer denatured protein are selected as candidates for western blot. Remaining candidates are further tested for specificity against other NMDAR subunits (GluN1/GluN2A, GluN1/GluN2B, GluN1/GluN2C, GluN1/GluN2D). (**B**) FSEC screening of the top six anti‐GluN3A antibody candidates using hybridoma supernatants and 10 nM GFP‐conjugated GluN1/GluN3A receptors. (**C**) Western blot analysis of GluN1/GuN3A receptors probed with anti‐GluN3A hybridoma supernatants. For comparison, GluN1/GluN3A receptors visualized with Coomassie Brilliant Blue (CBB) staining and detected with an anti‐GluN1 antibody are shown.

70 Perform an ELISA as in Support Protocol 1 with all positive hybridoma supernatants from the initial screening ELISA (see step 69), diluting supernatants 1:300 (Fig. 4A).

71 Simultaneously perform an ELISA as in Support Protocol 1 with the positive hybridoma supernatants from the initial screening ELISA (see step 69) using denatured protein, diluting supernatants 1:300. To denature the protein, dilute protein to 40 µg/ml in ELISA protein denature solution. Heat protein to 70°C for 30 min in a heat block. Dilute denatured protein to 2 µg/ml in TBS, 0.2% (v/v) Tween 20, and 0.2% BSA and add 50 µl/well to ELISA plate.
  Hybridoma supernatants positive at 1:300 dilution and preferring native over denatured antigen reflect candidate hybridomas to expand.
  If antibodies are desired for western blot or other denatured applications, the strongest positives that prefer denatured protein should also be expanded.

### Day 66: Subunit‐specificity ELISA

72 If needed, perform an ELISA as in Support Protocol 1 using proteins from various subunits that are not desired to rule out nonspecific binders.

### Day 66: Subculture candidate hybridomas

73 Immediately subculture the candidate hybridomas into 2 ml initial hybridoma growth medium per well in 24‐well tissue culture plates by gently pipetting them out of the 96‐well plates.

### Hybridoma characterization

74 Test the candidate hybridomas by FSEC for binding and protein shift, following the procedure outlined in step 14.
75 Test the candidate hybridomas by FSEC for super shift binding by mixing two candidate hybridomas with the protein, as described in step 14.
  A larger leftward shift indicates that both candidate hybridomas can bind to the protein simultaneously and may target unique binding sites (Fig. 4B).
76 Test the denatured candidate hybridomas by western blot (Fig. 4C).
77 When the cells reach confluence in 24‐well plates (see step 73), typically 5 to 10 days later, perform an ELISA as described in Support Protocol 1 using hybridoma supernatants diluted to 1:300, 1:900, 1:2700, and 1:8100.
  Test the IgG concentration in the supernatants using a commercial kit.
  Test the candidate hybridomas by Octet for subunit specificity and binding affinities (see Support Protocol 3).
78 Identify the hybridoma candidates to retain. Harvest each candidate from the 24‐well plate and centrifuge 10 min at 130 × *g*. Collect the supernatant for screening. Resuspend the cells in 1 ml medium and count them using trypan blue exclusion.
79 Freeze cells from each candidate well in 1 ml Opti‐MEM with 10% ultra‐low‐IgG FBS and 20% sterile DMSO in one 2‐ml cryogenic vial (1–2 × 10^5^ cells/ml). Place vial in a Mr. Frosty freezing container and freeze at –80°C overnight. The next day, transfer the frozen vials to a liquid nitrogen storage dewar for long‐term storage.
80 For each hybridoma candidate, set up cloning by limiting dilution in a 96‐well tissue culture plate with 0.5 cells in 200 µl per well.
81 Visually check the limiting dilution plates to carefully identify single cells forming monoclonal colonies.
82 After 10 to 14 days, when monoclonal colonies are well formed, pool supernatants from three best‐growing colonies and isotype the mixture using a mouse isotyping kit.
  A single heavy chain and light chain should be detected, confirming that all colonies are monoclonal.
83 If multiple isotypes appear, assay the supernatants individually to segregate out the isotypes and perform an antigen ELISA as described in Support Protocol 1 to determine which antibody is desired.
84 Subclone the monoclonal colonies into 2 ml medium per well in a 24‐well tissue culture plate.
85 When cells are confluent, expand to a T25 flask.
86 When cells are confluent, freeze down a vial as in step 79 and expand to a T75 flask.

## Support Protocol 1 DETECTION OF CONFORMATIONAL ANTIBODIES USING ELISA

This support protocol describes an ELISA method for screening serum and hybridoma supernatants (Basic Protocol 1) to detect conformation‐specific antibodies. The target iGluR antigen is immobilized on a 96‐well ELISA plate, and bound antibodies are detected using a secondary horseradish peroxidase (HRP)‐conjugated antibody and colorimetric ABTS substrate. The optical density at 405 nm indicates the antibody concentration and titer.

### Materials

3 µg/ml Strep‐Tactin (from 5 mg/ml Strep‐Tactin stock; see recipe) in TBS (see recipe)
TBS (see recipe)
ELISA blocking buffer (see recipe)
ELISA wash buffer (see recipe)
Target iGluR antigen (see Basic Protocol 1 and Fig. 2A)
ELISA dilution buffer (see recipe)
Serum (see Basic Protocol 1)
HRP–secondary antibody (HRP goat anti‐mouse IgG Fc, ImmunoChemistry Technologies, cat. no. 6292)
ABTS substrate (ABTS 2‐Component Microwell Peroxidase Substrate Kit, SeraCare, cat. no. 5120‐0032)
ELISA stop solution (see recipe)

96‐well ELISA plates (MaxiSorp, Thermo Fisher Scientific, cat. no. 44‐2404‐21)
Disposable reagent reservoirs (100 ml, VWR, cat. no. 89094‐658)
12‐channel pipettor
Plate seals (optional)
Plate reader (such as BMG CLARIOstar Plus)

*NOTE*: Unless otherwise mentioned, all incubation steps are performed stationary at 4°C or at any other temperature that is required to keep the protein stable.

1 Coat a 96‐well ELISA plate by adding 100 µl of 3 µg/ml Strep‐Tactin in TBS to each well using a disposable reagent reservoir and 12‐channel pipettor and incubating overnight at 4°C.
2 Gently wash the wells of the ELISA plate three times with 200 µl TBS per well.
3 Block the plate by adding 200 µl ELISA blocking buffer to each well and incubating overnight at 4°C or for 2 hr at room temperature.
  The plate can be stored sealed at 4°C for up to 1 week.
4 Gently wash the wells of the ELISA plate three times with 200 µl ELISA wash buffer per well.
5 Dilute the target iGluR antigen to 2 µg/ml with ELISA dilution buffer, add 50 µl to each well of the ELISA plate, and incubate at 4°C for 1 hr or seal the plate and incubate at 4°C overnight.
6 Gently wash the wells of the ELISA plate five times with 200 µl ELISA wash buffer per well.
7 Dilute the serum in ELISA dilution buffer at the following concentrations: 1:500, 1:1,000, 1:5,000, 1:25,000, 1:125,000, and 1:625,000.
8 Add 50 µl/well to the ELISA plate and incubate at 4°C for 1 hr.
9 Gently wash the wells five times with 200 µl ELISA wash buffer per well.
10 Dilute HRP–secondary antibody 1:5000 in ELISA dilution buffer. Add 100 µl/well to the ELISA plate and incubate at 4°C for 1 hr.
11 Gently wash the wells five times with 200 µl ELISA wash buffer per well.
12 Add 100 µl ABTS substrate per well to the ELISA plate. Watch wells for color development.
  Strong positives appear within 30 s, whereas weaker positives may take up to 10 min.
13 Stop the color reaction by adding 100 µl ELISA stop solution per well.
14 Read the optical density at 405 nm using a plate reader.

## Basic Protocol 2 EXPRESSION AND PURIFICATION OF MONOCLONAL ANTIBODIES AND THEIR DERIVATIVES

In this protocol, verified monoclonal hybridoma lines from Basic Protocol 1 are cultured in a bioreactor system to produce mAbs at milligram‐scale quantities. These hybridoma cells can be used to sequence the antibody VH and VL genes by standard RNA extraction either in house or through a commercial source. The sequences of the VH and VL genes are used to develop new antibody fragment constructs for expression of Fab or scFv in insect cells. Baculovirus generation and large‐scale Fab expression follow established protocols outlined in our previous publications (Goehring et al., 2014; Zhao et al., 2019) (Fig. 1C).

### Materials

Hybridoma cells (see Basic Protocol 1)
Hybridoma‐SFM (Thermo Fisher Scientific, cat. no. 12045076), 37°C
Hybridoma growth medium (see recipe), 37°C
Trypan blue (Corning/Cellgro, cat. no. 25‐900‐CL)
Protein A/G agarose (Pierce, cat. no, 20422)
Protein A IgG binding buffer (Pierce, cat. no. 21007)
Protein A IgG elution buffer (Pierce, cat. no. 21009)
1 M Tris buffer, pH 8.0
pFastBac Dual vector (Thermo Fisher Scientific, cat. no. 10712024)
*Escherichia coli* DH10 Bac cells (Life Technologies, cat. no. 10361‐012)
Bacmid selection plates (see recipe)
SF9 cells (Thermo Fisher Scientific, cat. no. 12659017)
SF900 III SFM (Thermo Fisher Scientific, cat. no. 1258027), 37°C
Sf9 Easy Titer cell line (Kerafast, cat. no. ENH022‐FP)
TBS (see recipe) supplemented with 5 mM desthiobiotin (IBA Lifesciences, cat. no. 2‐1000‐002)
Liquid nitrogen

Sorvall Legend XFR (Thermo Fisher Scientific, cat. no. 75004539)
CELLine bioreactor flask (Corning, cat. no. 353137)
Hemacytometer (Hausser Scientific, cat. no. 1492)
Inverted microscope (Nikon Eclipse TS100)
Econo‐Pac chromatography columns (Bio‐Rad, cat. no. 7321010)
50‐ml conical tubes
Flat‐bottom Erlenmeyer flasks (Greiner Bio‐One, cat. no. 679505)
27°C incubator shaker (Innova 44R; Eppendorf New Brunswick Scientific, cat. no. M1282‐0004)
Strep‐Tactin 4Flow high‐capacity resin (IBA Lifesciences, cat. no. 2‐1250‐010) equilibrated with TBS (see recipe)
30‐kDa‐cutoff filters (Amicon Ultra Centrifugal Filter, 30 kDa MWCO, Millipore Sigma, cat. no. UFC9030)
Superdex 200 Increase 10/300 GL column (Cytiva, cat. no. 28990944) equilibrated with TBS (see recipe)

Additional reagents and equipment for cell freezing (see Basic Protocol 1, step 79); cloning, transformation, and selection; PCR; Sf9 transfection and virus amplification (Goehring et al., 2014); endpoint dilution assay (Goehring et al., 2014); and supernatant collection and tangential flow filtration (see Support Protocol 2)

*NOTE*: Experiments involving PCR require extremely careful technique to prevent contamination.

### Monoclonal antibody production in a bioreactor flask

1 When hybridoma cells have reached 25–50 × 10^6^ cells, collect by centrifugation for 10 min at 130 × *g* in a Sorvall Legend XFR centrifuge. Collect and save the supernatant.
2 Add 1 L hybridoma‐SFM to the bulk medium chamber of the CELLine bioreactor flask.
3 Resuspend cells in 10 ml hybridoma growth medium and count by trypan blue exclusion using a hemacytometer and inverted microscope. Dilute the cells to 25–50 × 10^6^ cells total in 18 ml hybridoma growth medium and then add cells to cell compartment.
  When adding cells to the cell compartment, ensure that no air bubbles are introduced, as they can interfere with cell growth.
4 If seeding 25 × 10^6^ cells, culture in the bioreactor flask for 1 week until harvest. If seeding 50 × 10^6^ cells, culture for half a week. Continue culturing hybridomas in the bioreactor twice a week. Return 100 × 10^6^ cells in 18 ml to the cell compartment at each harvest and changing the bulk culture medium every third harvest. Freeze down at least three vials of cells as in Basic Protocol 1, step 79, at the first bioreactor harvest. Collect the supernatants from each harvest and freeze at –20°C until ready to purify antibody.
5 Pack 2 ml Protein A/G agarose beads into an Econo‐Pac chromatography column.
6 Thaw hybridoma supernatants from step 4 at 4°C and pool up to 45 ml.
7 Equilibrate the column with 20 ml Protein A IgG binding buffer.
8 Dilute sample at least 1:1 with the binding buffer before application to the Protein A/G column to maintain the proper ionic strength and pH for optimal binding. If culture medium becomes hazy upon dilution with the binding buffer, centrifuge the diluted sample for 20 min at 4000 × *g*.
9 Apply the diluted sample to the column and save the flow‐through in case the total amount of antibody exceeds the column capacity.
10 Wash the column with 20 ml binding buffer.
11 Elute antibodies with 18 ml Protein A/G IgG elution buffer into a 50‐ml conical tube. Immediately adjust the sample to physiologic pH using 1 M Tris buffer (pH 8.0).
  The pH can be adjusted by adding 2 ml of 1 M Tris buffer, pH 8.0, to the empty conical tube prior to elution so the pH is adjusted during elution.
12 Regenerate the column by washing with another 5 to 10 ml elution buffer. Wash the column with 20 ml binding buffer and store at 4°C for reuse.
  Columns may be regenerated at least 10 times without significant loss of binding capacity.

### Antibody fragment expression, purification, and size‐exclusion chromatography separation

13 Clone the heavy‐ and light‐chain antibody gene sequences into the pFastBac Dual vector.
14 Transform the pFastBac Dual construct into *E. coli* DH10 Bac cells to generate recombinant bacmid DNA. After transformation, perform blue‐white colony selection using a bacmid selection plate. Isolate white colonies and re‐streak them on a fresh bacmid selection plate. Confirm antibody gene sequence insertion using PCR.
15 Transfect Sf9 cells in SF900 III SFM with recombinant bacmid DNA to generate P1 virus and amplify the virus by infecting fresh Sf9 cells to generate P2 virus stock, following the established procedure (Goehring et al., 2014).
16 Determine the titer of the P2 recombinant baculovirus using the Sf9 Easy Titer cell line and the endpoint dilution assay (Goehring et al., 2014).
17 Prepare the Sf9 cells ∼10 days in advance of infection (see steps 18 and 19) using a flat‐bottom Erlenmeyer flask to reach the density of 1.5–2.5 × 10^6^ cells/ml prior to the infection. Determine the total number of cells and percent viability using trypan blue exclusion.
18 Add recombinant P2 baculovirus at an MOI of 1 to 5 to infect 3 L of Sf9 cells at a density of 1.5–2.5 × 10^6^ cells/ml.
  The volume of virus suspension added should not exceed >10% of the culture volume.
19 Collect the supernatant after 96 to 120 hr of transduction at 27°C (see Support Protocol 2).
  The incubation time and temperature for baculovirus‐transduced Sf9 cells should be determined separately for each Fab.
20 Concentrate the supernatant to ∼200 ml using tangential flow filtration using 30‐kDa‐cutoff filter (see Support Protocol 2).
  An alternative method for purifying Strep‐tagged Fab proteins from large volumes of cell culture supernatant is the WET FRED technique (WET FRED Set, IBA Lifesciences, cat. no. 2‐0910‐001). This method employs gravitational flow columns and reduces the biotin concentration in the cell culture medium with the BioLock solution (IBA Lifesciences, cat. no. 2‐0205‐050).
21 Mix the filtered supernatant with Strep‐Tactin 4Flow high‐capacity resin equilibrated with TBS for 1 hr at 4°C.
22 Wash the resin with 10 column volumes of TBS to remove contaminants.
23 Elute the Fab from column using TBS supplemented 5 mM desthiobiotin.
24 Concentrate the eluted sample using 30‐kDa‐cutoff filter to ∼1 ml final volume.
25 Perform SEC purification by injecting ∼1 ml sample onto Superdex 200 Increase 10/300 GL column equilibrated with TBS and collect the eluted peak fractions.
26 Pool the fractions, concentrate them using a 30‐kDa‐cutoff filter, and aliquot the samples. Plunge‐freeze the aliquots in liquid nitrogen and store at –80°C.
  Assess the binding affinities of the generated mAbs and Fabs using BLI (see Support Protocol 3) and evaluate subunit specificity, cross‐reactivity, and competition dynamics using FSEC (see Basic Protocol 1, step 14) (Kawate & Gouaux, 2006).
  Purified Fabs can be stored at –80°C for months before use.

## Support Protocol 2 CONCENTRATION AND CLARIFICATION OF INSECT CELL SUPERNATANT FOR Fab PURIFICATION

This support protocol describes the tangential flow filtration process for concentrating 3 L Fab protein expressed in insect cell culture supernatant (Basic Protocol 2). The procedure aims to reduce the supernatant processing volume and remove biotin and macromolecular impurities smaller than 30 kDa, thereby optimizing the efficiency of downstream affinity purification. The method is scalable based on the initial volume, with all steps performed at room temperature.

### Materials

Sf9 cell culture (see Basic Protocol 2, step 19)
50 mM unbuffered Tris base (Fisher Scientific, cat. no. BP152)
Distilled water
0.1 and 0.5 M NaOH solutions (see recipes; make fresh)
TBS (see recipe)

Magnetic stir plate and stir bar
Avanti JXN centrifuge, 4°C
0.2‐µm nitrocellulose membrane filters
T‐Series cassettes with Omega PES membrane, 30‐kDa filter (Cytiva, cat. no. OS030T12)
Centramate tangential flow concentrator (with Masterflex Easy Load II tangential flow pump; Pall, Cytiva)
2 L Ultralab system (Cytiva, cat. no. FS006 × 75)
Optima MAX‐XP tabletop ultracentrifuge (Beckman, cat. no. 393315), 4°C

Additional reagents and equipment for affinity purification (see Basic Protocol 2, steps 13 to 26)

1 Harvest 3 L cell culture supernatant from Sf9 cell culture (see Basic Protocol 2, step 19) and add 50 mM unbuffered Tris base.
2 Stir the samples for 30 min at room temperature on a magnetic stir plate with a stir bar to adjust the pH to 8.0.
  A significant amount of precipitation should form.
3 Collect the supernatant by centrifugation using an Avanti JXN centrifuge for 30 min at 6000 × *g*, 4°C.
4 Filter the supernatant twice using a 0.2‐µm nitrocellulose membrane filter.
5 Assemble T‐Series cassettes with Omega PES membrane (30‐kDa filter) into the gasket of Centramate tangential flow concentrator, connected to a Masterflex Easy Load II tangential flow pump and a 2 L Ultralab system. Set the pump rate to 120 rpm.
6 Prior to use, cleanse the filter/tubing by adding 1 L distilled water into the assembled 2 L Ultralab system, using the knob to direct the flow toward the wash while positioning the tubes in the waste collector.
7 Wash the system with 1 L fresh 0.5 M NaOH solution, followed by 1 L fresh 0.1 M NaOH solution, 1 L distilled water, and finally with 1 L TBS. Direct the flow‐through in the waste.
8 Add filtered supernatant from step 4 to the chamber and turn the knob towards the chamber so that the flow is directed towards concentrate.
9 Concentrate the samples to 200 ml and then add 1 L TBS into the chamber and concentrate again to 200 ml. Repeat the process three times total.
10 Collect the final ∼200 ml supernatant and centrifuge 40 min at 86,500 × *g*, 4°C, in an Optima MAX‐XP tabletop ultracentrifuge.
11 Filtrate the supernatant using a 0.2‐µm nitrocellulose membrane filter and subsequently employ supernatant for affinity purification (see Basic Protocol 2, steps 13 to 26).
  We recommend using a Strep tag, Twin‐Strep tag, or His tag for efficient affinity purification (see Table 4 for a list of available in‐house construct designs).
12 Wash the system using the reverse order of sequential washing outlined in step 7.

## Support Protocol 3 MEASUREMENT OF IONOTROPIC GLUTAMATE RECEPTOR BINDING KINETICS USING OCTET BLI SYSTEM

This protocol outlines the procedure for measuring the kinetics of binding of iGluRs to in‐house‐developed mAbs (Basic Protocol 2) using the Octet BLI System (Sartorius) with anti‐mouse IgG Fc capture (AMC) biosensors. The measurement protocol follows the manufacturer's guidelines, with optimizations of buffer composition and antibody concentrations.

### Materials

Octet Anti‐Mouse Fc Capture (AMC) Biosensors (Sartorius, cat. no. 18‐5088)
TBS (see recipe) containing 0.075% (w/w) LMNG (Anatrace, cat. no. NG310) or 1 mM DDM (Anatrace, cat. no. D310)
10 mM glycine buffer, pH 1.7 (Sigma, cat. no. G8898)
25 µg/ml purified mAb solution (see Basic Protocol 2)
TBS (see recipe)
iGluR analytes at varying concentrations

Octet RED384 System (ForteBio)
ForteBio Octet Data Analysis HT software

1 Hydrate Octet AMC Biosensors in TBS containing 0.075% (w/w) LMNG or 1 mM DDM for 10 min at room temperature.
2 Perform three conditioning cycles by immersing biosensors in 10 mM glycine buffer (pH 1.7) for 20 s and transferring them to TBS‐containing detergent solution for 20 s.
3 Incubate biosensors in 25 µg/ml purified mAb solution for 600 s.
4 Equilibrate mAb‐coated biosensors in TBS for 300 s.
5 Transfer mAb‐coated biosensors to wells containing iGluR analytes at varying concentrations, ensuring the highest analyte concentration is set at 10 to 20× the expected K_D_.
6 Measure association kinetics for 900 s using the Octet RED384 System.
7 Measure dissociation kinetics for 1800 s, including in at least one reference well without analyte to correct for background signals.
8 Use ForteBio Octet Data Analysis HT software to subtract reference biosensor signals, align *y*‐axis to the Baseline step, apply Savitzky‐Golay filtering, and fit data to a 1:1 binding model using “full” global fitting.
9 Calculate kinetic constants and average them across all sample concentrations with valid curve fits.
10 Present binding kinetics data by displaying representative binding curves and corresponding kinetic constants.

## REAGENTS AND SOLUTIONS

### Bacmid selection plates

2% (w/v) Luria‐Bertani (LB) agar, Miller (BD, cat. no 244520)
50 µg/ml kanamycin (Sigma, cat. no. K0879)
10 µg/ml tetracycline (Sigma, cat. no. T7660)
7 µg/ml gentamicin (Sigma, cat. no. G1264)
100 µg/ml Bluo‐Gal (Invitrogen, cat. no. 15519028)
40 µg/ml IPTG (Anatrace, cat. no. I1003)
Pour into plates
Store ≤1 month at 4°C

### CpG oligonucleotide, 2 mg/ml

Resuspend lyophilized power (Invivogen, cat. no. tlrl‐1668) to 2 mg/ml in sterile endotoxin‐free water and aliquot. Store ≤1 year at –20°C.

### ELISA blocking buffer

20 mM Tris‐HCl, pH 8 (Fisher Scientific, cat. no. BP152)
150 mM NaCl (Fisher Scientific, cat. no. BP358)
3% (w/v) BSA (Fisher Scientific, cat. no. BP1600)
Store ≤1 week at 4°C

### ELISA dilution buffer

20 mM Tris‐HCl, pH 8 (Fisher Scientific, cat. no. BP152)
150 mM NaCl (Fisher Scientific, cat. no. BP358)
0.2% (w/v) BSA (Fisher Scientific, cat. no. BP1600)
0.5 mM DDM (Anatrace, cat. no. D310)
Store ≤1 week at 4°C
  The detergent should be the same as that the protein was purified with for protein stability.

### ELISA protein denature solution

20 mM Tris‐HCl, pH 8 (Fisher Scientific, cat. no. BP152)
150 mM NaCl (Fisher Scientific, cat. no. BP358)
8 M urea (Millipore Sigma, cat. no. U1250)
1% (v/v) 2‐mercaptoethanol (Sigma, cat. no. M6250)
Prepare fresh immediately before use

### ELISA stop solution

20 mM Tris‐HCl, pH 8 (Fisher Scientific, cat. no. BP152)
150 mM NaCl (Fisher Scientific, cat. no. BP358)
1% (w/v) sodium dodecyl sulfate (SDS; Bio‐Rad, cat. no. 1610303)
Store ≤1 month at room temperature

### ELISA wash buffer

20 mM Tris‐HCl, pH 8 (Fisher Scientific, cat. no. BP152)
150 mM NaCl (Fisher Scientific, cat. no. BP358)
0.5 mM DDM (Anatrace, cat. no. D310)
Store ≤1 week at 4°C
  The detergent should be the same as that the protein was purified with for protein stability.

### FSEC buffer

20 mM Tris (Fisher Scientific, cat. no. BP152)
150 mM NaCl (Fisher Scientific, cat. no. BP358)
0.075% (w/w) LMNG (Anatrace, cat. no. NG310)
Store ≤1 week at 4°C

### HAT selection medium

183.5 ml Opti‐MEM I (Thermo Fisher Scientific, cat. no. 31985088)
33.75 ml ultra‐low‐IgG FBS (Thermo Fisher Scientific, cat. no. 16250078)
1.25 ml Hybridoma Cloning Factor (Millipore Sigma, cat. no. 11363735001)
4.5 ml HAT (made up in 10 ml Opti‐MEM I immediately prior to use; Millipore Sigma, cat. no. H0262)
2.25 ml 100× antibiotic antimycotic (Millipore Sigma, cat. no. A5955)
Prepare fresh immediately before use
  This recipe is for one spleen, processed in eight 96‐well plates.

### Hybridoma growth medium

500 ml Opti‐MEM I (Thermo Fisher Scientific, cat. no. 31985088)
50 ml ultra‐low‐IgG FBS (Thermo Fisher Scientific, cat. no. 16250078)
Store ≤1 month at 4°C

### Initial hybridoma growth medium

500 ml Opti‐MEM I (Thermo Fisher Scientific, cat. no. 31985088)
50 ml ultra‐low‐IgG FBS (Thermo Fisher Scientific, cat. no. 16250078)
5 ml 100× antibiotic antimycotic (Millipore Sigma, cat. no. A5955)
2.5 ml Hybridoma Cloning Factor (Millipore Sigma, cat. no. 11363735001)
Store ≤1 month at 4°C

### Monophosphoryl lipid A (MPLA), 1 mg/ml

Resuspend lyophilized powder (Invivogen, cat. no. vac‐mpla) to 1 mg/ml in DMSO (Millipore Sigma, cat. no. D2650) and aliquot. Store ≤1 year at –20°C.

### Myeloma growth medium

500 ml RPMI medium (ATCC, cat. no. 30‐2001)
50 ml FBS (Thermo Fisher Scientific, cat. no. 10082147)
Store ≤1 month at 4°C

### NaOH solution, 0.1 M

Dissolve 8 g NaOH (Sigma, cat. no. 221465) in 2 L distilled water. Prepare fresh immediately before use and allow the solution to reach room temperature.

### NaOH solution, 0.5 M

Dissolve 40 g NaOH (Sigma, cat. no. 221465) in 2 L distilled water. Prepare fresh immediately before use and allow the solution to reach room temperature.

### Strep‐Tactin, 5 mg/ml

Resuspend lyophilized powder (IBA, cat. no. 2‐1204‐001) in sterile 50% (v/v) glycerol in water. Store ≤1 year at –20°C.

### Tris‐buffered saline (TBS)

20 mM Tris‐HCl, pH 8 (Fisher Scientific, cat. no. BP152)
150 mM NaCl (Fisher Scientific, cat. no. BP358)
Filter using 0.22‐µm membrane filter (Fisher Scientific, cat. no. 09‐719‐2B)
Store ≤1 month at room temperature

## COMMENTARY

### Background Information

Conformation‐specific antibodies are generated to recognize native, 3D epitopes on integral MPs, which are difficult to study due to their amphipathic and dynamic nature. These antibodies target conformational epitopes that are present in the functional state of the protein. They are crucial for structural studies, such as cryo‐EM and X‐ray crystallography, where they stabilize specific conformations for high‐resolution structural analysis. Additionally, they are used in biochemical assays like ELISA, flow cytometry, and western blotting. Our approach is specifically designed to raise antibodies against 3D epitopes of MPs. This strategy distinguishes itself through unique antigen preparation; distinct liposome formulation, reconstitution, and immunization methods; and the detection of 3D epitope–binding antibodies using ELISA and FSEC techniques.

### Critical Parameters and Troubleshooting

#### Antigen preparation and stability

To achieve a successful immune response, MPs must retain their native fold and functional activity during solubilization and reconstitution into liposomes (Basic Protocol 1). If MPs lose activity, optimizing the lipid composition or selecting the appropriate detergent is critical. Adjusting the detergent concentration and buffer conditions (such as pH and ionic strength) can further enhance protein stability. Troubleshooting (see Table 1) may involve modifying lipid ratios, switching to alternative detergents, or adding stabilizing agents to preserve the functionality of MPs.

**Table 1:** Troubleshooting Guide for Generation of Conformation‐Specific Monoclonal Antibodies for Integral Membrane Proteins

Problem	Possible cause	Solution
Poor protein incorporation into liposomes	Improper liposome preparation	Optimize extrusion conditions by adjusting membrane pore size, number of passes, and pressure to achieve uniform liposome size. Alternatively, use sonication or controlled freeze‐thaw cycles to break down large liposomes effectively.
	Protein/lipid ratio is off	Adjust protein/lipid ratio to determine desirable ratio to ensure efficient incorporation.
	Protein instability in the detergent solution	Change to a milder detergent with lower critical micelle concentration or test detergent mixtures to enhance stability. Add stabilizing agents like cholesterol, lipids, or small‐molecule chaperones to enhance protein stability. The same detergent used to purify the protein should be used for incorporation into liposomes. Screening may be required to find the optimal detergent for protein stability.
	Incomplete detergent removal	Increase amount of bio‐beads or perform sequential incubations with fresh beads to enhance detergent removal. Optimize incubation time, gentle agitation, and temperature to maximize bead efficiency.
Low serum titer	Poor proteoliposome quality	Optimize lipid composition and reconstitution methods to improve proteoliposome quality. Vary protein/lipid ratio to increase protein incorporation and ensure proper protein incorporation. Validate protein integrity and use suitable adjuvants to boost immune response.
	Weakly immunogenic antigen	Increase protein incorporation into liposomes and antigen dose. Use additional immunizations if serum titer continues to increase. Alternatively, try different mouse strains, such as a knockout of your antigen.
Contamination of cell culture	Change in medium color and/or foul smell	Ensure that proper sterile technique is used. Clean the laminar flow hood with 70% (v/v) ethanol prior to use. Clean all items transferred into the hood with 70% ethanol before use.
Poor Fab expression	Low‐quality cells	Do not let cells overgrow and subculture on a regular basis. Do not use cells that are more than 30 passages since being raised from liquid nitrogen.
	Wrong MOI	Screen different MOI values to determine optimal expression.
	Wrong expression conditions	Screen expression at different incubation times and temperatures to determine optimal expression.

#### Spleen injection

This method employs targeted PL antigen delivery to the spleen during final immunization (Basic Protocol 1), efficiently activating both innate and adaptive immune responses and thereby increasing the probability of producing activated B cells needed for hybridoma fusion. This procedure necessitates precise surgical technique and must be carried out under strict aseptic conditions to prevent contamination and ensure effective activation of the immune system.

#### Antibody screening

Antibody screening using ELISA and FSEC (Basic Protocol 1 and Support Protocol 1) effectively identifies high‐specificity 3D epitope binders. To address poor specificity in ELISA (see Table 1), optimize antigen concentration, incubation times, and washing conditions to reduce nonspecific binding. In FSEC, improve resolution and minimize nonspecific interactions by adjusting detergent concentration, sample load, and buffer conditions.

### Understanding Results

Basic Protocol 1 describes the generation of conformation‐specific mAbs against iGluRs. Using this protocol, mAbs were generated against seven iGluR subunit complexes and three auxiliary proteins, as detailed in the Supporting Information, Table S1. Typically, for immunization, the iGluR protein is solely fused with a carboxy‐terminal Strep tag II for affinity purification, with no fluorescent protein tag, as we have found that the presence of a fluorescent protein tag dominates the immune response. iGluR proteins were purified using affinity chromatography and subsequent SEC, with the resulting SEC profile and SDS‐PAGE analysis of the GluN1/3A NMDAR complex shown in Figure 2A.

Our standard liposome preparation includes asolectin, cholesterol, brain polar lipid extract, and MPLA at a 3.5:1:0.2:1 ratio. Lipids are dissolved in organic solvents, subjected to multiple freeze‐thaw‐sonication cycles, and extruded through polycarbonate filters to generate unilamellar liposomes (Fig. 2B). Antigen incorporation into liposomes is achieved via detergent disruption or liposomal solubilization methods at protein/lipid ratios of 1:50 to 1:100, as detailed in Basic Protocol 1, steps 11 to 16. Excess detergent is removed using bio‐beads, and PL incorporation efficiency is assessed via SDS‐PAGE with standards (Fig. 2C). Alternatively, the FSEC method can be used to assess both antigen integrity and PL incorporation efficiency (Kawate & Gouaux, 2006). PLs that are fully loaded with antigen, in our experience, give rise to the most robust immune response and the highest likelihood of desired antibody generation. Therefore, we want at least half of the protein initially solubilized to be incorporated into the liposomes.

The immunization schedule, dosage, adjuvants, and administration methods are detailed in Basic Protocol 1, steps 17 and 18 (Fig. 1A and 1B). PL‐reconstituted antigens are administered intraperitoneally at a dose of 25 µg per mouse and are generally effective in inducing an immune response. The adjuvant formulation includes MPLA and class B cytosine‐phosphate‐guanine‐motif oligonucleotides, with an optimized dose that induces a strong immune response while limiting animal distress and pain.

To ensure effective B‐cell activation, the PL administration site needs to be positioned approximately 3 to 5 mm lateral to the midline and just above hip level on the left side of the mouse to promote antigen draining to the spleen (Fig. 3A). Two intraperitoneal injections are given 2 weeks apart, and the serum titer is tested 12 days later. If the serum titer is low, a third injection is given 1 week after the blood draw, and the serum is tested again after 12 days. If a sufficient increase in the serum titer is not achieved after a third immunization, the project is typically stopped, and other strategies are considered. Mice with sufficient serum titers are rested for 30 days from the last immunization before the final immunization and fusion.

To determine the level of immune response, based on serum titer, and to detect antibodies that bind to conformational epitopes, we use an affinity‐tag capture ELISA. The ELISA wells are coated with a streptavidin variant, Strep‐Tactin, allowing capture of the purified, Strep tag–tagged protein in a native conformation. Antigen with a fluorescent protein tag such as GFP is also acceptable for the ELISA screening. A mild detergent such as DDM, LMNG, or digitonin is used in the ELISA buffers, and the assay is carried out at 4°C to promote protein stability. Serum from immunized mice is diluted from 1:500 to 1:625,000, and an HRP anti‐mouse IgG secondary antibody is used for detection (Support Protocol 1), as shown in Figure 3B. A high serum titer is the best indicator that a hybridoma fusion will produce antibodies of interest. We expect to see a serum titer beyond 1:25,000 dilution for a strong immune response; for GluN1/GluN3A‐immunized mice, a serum titer of nearly 1:625,000 was observed (Fig. 3C). Furthermore, serum is mixed with antigen and evaluated by FSEC to detect binding; in the case of GluN1/GluN3A‐immunized mice, a strong peak shift indicating multiple bound antibodies was observed (Basic Protocol 1, steps 21 to 23) (Fig. 3D).

Even if mice have robust serum titers, this does not guarantee the generation of hybridomas secreting conformation‐specific antibodies following fusion. Thus, to enhance the probability of production of conformation‐specific antibodies, we carry out a final boost to induce a strong, short‐lived immune response that activates immune cells in the lymphoid organ that will be used for the fusion. Therefore, we chose to deliver the final boost of 10 µg antigen without adjuvant, so as not to affect spleen cell function, directly into the spleen of the highest‐serum‐titer mice (Basic Protocol 1, steps 24 to 38) (Gearing et al., 1985; Ove Nilsson & Larsson, 1990). Administering MP antigen directly into the spleen is technically challenging and requires surgical expertise. However, it significantly stimulates the activation of antibody‐producing B cells needed for hybridoma fusion. Three days after the final immunization, the spleens are harvested, and a classical PEG fusion is performed (Basic Protocol 1, steps 44 to 67) (Galfre et al., 1977).

Supernatants from all wells of the hybridoma fusion are first tested with an ELISA using native antigen. The top hits are chosen and tested again at lower dilution on native and denatured antigen, and any hits that prefer native over denatured antigen by at least two‐fold at this step are considered as possible conformation‐specific antibodies of interest (Basic Protocol 1, steps 68 to 72). These candidates can be further screened to ensure specificity. As an example, for the GluN3A candidates, we screened the initial hits against NMDARs composed of non‐GluN3A subunits (Fig. 4A), thus allowing us to discover subunit‐selective and subunit‐generic antibodies, the latter of which are often useful. Subsequently, we could assess hybridoma supernatants for potential affinity to the folded protein through ELISA at greater dilution. Top candidates are further screened by an FSEC shift assay to confirm binding with the native antigen, showing strength of shift to void and epitope recognition. Additionally, potential linear binders identified through ELISA screening with denatured protein can be further validated using western blot analysis.

For the GluN1/GluN3A receptor, the initial ELISA screening identified 34 candidate hybridoma supernatants. A subsequent cross‐reactivity assessment with other NMDAR subunits, including GluN1 and GluN2A‐D, excluded 18 candidates due to their cross‐reactivity. Pairwise binding tests further narrowed the selection by eliminating antibodies with overlapping or closely situated epitopes, resulting in six candidates with distinct binding sites: 1E8, 2D3, 2G4, 3G9, 5E3, and 6G8 (Fig. 4B). Western blot analysis revealed that 2G4 recognizes a linear epitope on the GluN3A subunit, whereas 1E8, 2D3, 3G9, and 5E3 are conformation specific (Fig. 4C). Positive candidate hybridomas are subcultured and saved during the characterization process. Once candidates have been finalized, they are cloned by limited dilution to ensure monoclonality, and the monoclonal lines are then retested for the production of the desired mAb (Basic Protocol 1, steps 78 to 86).

The iGluR‐specific mAbs we have generated are summarized in Table 2. These mAbs were systematically evaluated by FSEC shift assays to determine their binding specificity to the native receptor, competitive binding dynamics, and cross‐reactivity with other subunits. The GluN1/N2A receptor was incubated with the NMDAR‐specific mAbs 5F11, 3D2, 6E9, and 5E3, individually and in combination, at a 1:2 molar ratio. The [5F11: GluN1/N2A] complex exhibited a stronger left shift compared to the [3D2: GluN1/N2A] complex. Incubation of GluN1/N2A with all four NMDAR‐specific mAbs resulted in a shift identical to that observed with the 5F11 mAb, confirming its target specificity and a distinct binding site that remains unperturbed by the presence of other mAbs (see Supporting Information, Fig. S1A). Similarly, other mAbs were tested with their target receptor and resulted in an FSEC shift toward the void and binding to the corresponding epitope without competition (see Supporting Information, Fig. S1B‐H). The binding test showed that the 9B5 mAb specifically targets TARP γ‐8, whereas the 4B2 mAb interacts with TARP γ‐2 and has modest cross‐reactivity with TARP γ‐8. The non‐overlapping epitopes of 4B2 and 9B5 on TARP γ‐2 were confirmed by the [4B2: TARP γ‐2:9B5] complex exhibiting a greater leftward shift than the [4B2: TARP γ‐2] complex, indicating simultaneous binding of the two mAbs. The 2H1 mAb displayed specificity for CNIH2, whereas it did not bind to its homologous target, CNIH3 (see Supporting Information, Fig. S1I‐L). Cross‐reactivity was assessed using higher concentrations of AMPAR‐specific mAbs. The 11B8 and 15F1 mAbs were found to be specific to GluA1 and GluA2 AMPARs, respectively. In contrast, the 5B2 mAb exhibited cross‐reactivity with GluA4 AMPARs at ratios of 1:5 and 1:15 (see Supporting Information, Fig. S2).

**Table 2:** Summary of In‐House‐Generated Monoclonal Antibodies Targeting iGluRs*^a^*

Ab	Target specificity	Isotype	Species reactivity	Binding epitope	Western blot	FSEC shift assay	In‐house availability	Competition
5F11	GluN1‐specific	IgG1/kappa	XRM	N/A	Negative	Positive	HBC, FabC	Compatible
3D2	GluN2A‐specific	IgG1/kappa	RM	N/A	Negative	Positive	HBC, FabC	Compatible
6E9	GluN2D‐specific (mAb exhibits weak cross‐reactivity with GluN2A, whereas Fab does not)	IgG2a/kappa	RM	N/A	Very weakly positive	Positive	HBC	Compatible
5E3	GluN3A‐specific	IgG1/kappa	RM	N/A	Negative	Positive	HBC, FabC	Compatible
3G9	GluN3A‐specific	IgG2a/kappa	RM	N/A	Negative	Positive	HBC, FabC	Compatible
2D3	GluN3A‐specific	IgG2b/kappa	RM	N/A	Negative	Positive	HBC, FabC	Compatible
11B8	GluA1‐specific	IgG2b/kappa	RM	ATD	Positive	Positive	HBC, FabC, ScFvC	Compatible
15F1	GluA2‐specific	IgG1/kappa	RMHPSC	ATD	Positive	Positive	HBC, FabC, ScFvC	Compatible
5B2	GluA3‐specific but also weakly reacts with GluA4	IgG1/kappa	RM	ATD	Positive	Positive	HBC, FabC	Compatible
4B2	TARP γ‐2, but also binds to γ‐8	IgG2a/kappa	R	C‐terminal 40‐aa segment	Negative	Positive	HBC, FabC	Compete with 9B5
9B5	TARP γ‐8‐specific	IgG2a/kappa	R	N/A	Positive	Positive	HBC, FabC	Compatible
13A8	TARP γ‐8‐specific	IgG2a/kappa	R	N/A	Positive	Positive	mAb	Compatible
15G7	TARP γ‐8‐specific	IgG2a/kappa	R	N/A	Positive	Positive	HBC	Compatible
13C3	TARP γ‐8‐specific	N/A	R	N/A	Positive	Weakly positive	HBC	N/A
13F7	TARP γ‐8‐specific	N/A	R	N/A	Positive	Positive	HBC	N/A
2H1	CNIH2‐specific	IgG2a/kappa	RM	Intracellular TM2‐TM3 loop	Positive	Positive	HBC	Compatible

^*a*^ Abbreviations: N/A – Not assessed, HBC – Hybridoma cells, FabC – Fab gene construct, ScFvC – ScFv gene construct in the pFastBac vector. Species reactivity validated experimentally includes X – Xenopus, R – Rat, M – Mouse, H – Human, P – Pig, S – Sheep, and C – Cow.

The kinetics of mAbs binding to their target receptor were characterized using BLI (Yang et al., 2017). The association (k_on_) and dissociation (k_off_) rates were measured to determine equilibrium dissociation constants (K_D_) (Support Protocol 3), with the results for each iGluR mAb summarized in Table 3 and presented in the Supporting Information, Figure S3. All mAbs exhibited sub‐nanomolar (nM) affinities, characterized by rapid association rates and exceptionally slow dissociation rates. The robust signal observed during the association phase likely reflects strong binding to the target epitope. The observed fast on rates and extremely slow off rates reflect the high affinity and stability of the iGluR‐mAb complexes. These complexes exhibited prolonged binding interactions, with dissociation rates so slow that they reached the detection limits of the BLI system.

**Table 3:** Binding Kinetics of iGluR Antibodies Assessed Using Octet RED384*^a^*

Antibodies	Targeting antigen	K_D_ (nM)	k_on_ (Ms^−1^)	k_off_ (S^−1)^
5F11 mAb	GluN1	0.43	1.42E+05	6.19E‐05
3D2 mAb	GluN2A	<1.0E‐12	1.85E+06	<1.0E‐07
6E9 mAb	GluN2D	<1.0E‐12	1.45E+08	<1.0E‐07
5E3 mAb	GluN3A	1.40	8.37E+04	1.99E‐05
11B8 mAb	GluA1	<1.0E‐12	1.23E+05	<1.0E‐07
15F1 mAb	GluA2	<1.0E‐12	1.02E+06	<1.0E‐07
5B2 mAb	GluA3	<1.0E‐12	7.58E+05	<1.0E‐07
4B2 mAb	TARP γ‐2	4.5	5.03E+04	1.19E‐04
9B5 mAb	TARP γ‐8	3.1	4.59E+04	1.03E‐03
2H1 mAb	CNIH2	<1.0E‐12	1.55E+04	<1.0E‐07

^*a*^ The equilibrium binding constants (K_D_), association rates (k_on_), and dissociation rates (k_off_) were determined using ForteBio Octet RED384. Data were fitted to Langmuir binding kinetics to analyze the interaction between immobilized iGluR mAbs and their target receptor.

In many instances, the most successful utilization of antibodies and antibody fragments benefits from determination of their binding epitopes to the target antigen. iGluRs are composed of the amino‐terminal (ATD/NTD), ligand‐binding (LBD), transmembrane (TMD), and C‐terminal (CTD) domains (Yu et al., 2021). To achieve this, we employed cryo‐EM structure analysis of antigen‐antibody complexes, overlapping peptide library screening, and FSEC‐based epitope mapping methods. The cryo‐EM structure of the antibody‐bound AMPAR complex revealed that the epitope sites for the 11B8, 15F1, and 5B2 antibodies are located within the ATD of the AMPAR, with interactions distributed throughout secondary structure elements. The epitope residues are not conserved among subunits, indicating that the antibodies bind to subunit‐specific epitopes (Yu et al., 2021). The GluA2 AMPAR sequences among human, pig, sheep, and cow revealed residue conservation, suggesting that the GluA2‐specific 15F1 antibody is a useful tool for uncovering GluA2‐containing complexes in many mammals (Rao & Gouaux, 2023). The 4B2 mAb epitope site was confirmed by a peptide competition assay, revealing its proximity to the transmembrane region (between residues 219 and 234) of the TARP γ‐2 C‐terminus. The epitope‐binding site of the 2H1 mAb was identified using the FSEC peptide competition method, located between C‐terminal residues 99 and 118 of CNIH2.

We succeeded in obtaining several conformational antibodies targeting various ion channels and transporter proteins to facilitate native purification and structural studies. Once native‐folded antigen has been prepared, the time it takes to obtain mAb candidates can range from 90 to 120 days depending on the immune response of the mice and cell growth rates. The overall process from PL preparation to recombinant Fab production takes ∼4 months. This set of protocols allowed us to successfully develop conformation‐specific antibodies that bind to iGluRs. The identified VL and VH sequences are presented in the Supporting Information in Table S2 and are valuable resources for the future experiments. In‐house‐available iGluR‐specific antibody fragment constructs are listed in Table 4. In many instances, the in‐house‐generated antibodies show nM to sub‐nM affinity, fast on and slow off rates, high specificity, long‐term stability, and promise for large‐scale expression and subsequent chemical modification. The developed mAbs, scFvs, and Fabs against iGluRs are powerful tools for scientists across a broad swath of structural biology and neuroscience.

**Table 4:** List of In‐House Antibody Derivative Plasmids Targeting iGluRs*^a^* *^,^* *^b^*

S. no	Antigen	Plasmid code	Construct design description
1	GluN1	5F11‐STT	Fab, SP‐LC; SP‐HC‐TS‐STII‐pFBd
2		5F11‐CST	Fab, SP‐LC; SP‐HC‐FC‐TS‐ STII‐pFBd
3		5F11‐GHS	Fab, SP‐LC; SP‐HC‐TS‐GFP‐8×HT‐STII‐pFBd
4		5F11‐HST	Fab, SP‐LC; SP‐HC‐TS‐halo tag‐ STII‐pFBd
5		5F11‐mCS	Fab, SP‐LC; SP‐HC‐TS‐mCherry‐STII‐pFBd
6		5F11‐GHF	Fab, SP‐LC; SP‐HC‐TS‐GFP‐8× HT‐3×FT‐pFBd
7	GluN2B	2B5‐CST*^c^*	Fab, SP‐LC‐TS‐8×HT‐L‐8×HT‐TS‐HC‐FC‐TEV‐2×STII‐pFBd
8		2B5‐GST*^c^*	Fab, SP‐LC‐TS‐8×HT‐L‐TS‐HC‐FC‐TEV‐GFP‐3C‐2×STII‐pFBd
9	GluN3A	5E3‐GFT	Fab, SP‐LC; SP‐HC‐TS‐TEV‐GFP‐8×HT‐3C‐3×FT‐pFBd
10		3G9‐GFT	Fab, SP‐LC; SP‐HC‐TS‐TEV‐GFP‐8×HT‐3C‐3×FT‐pFBd
11		2D3‐GFT	Fab, SP‐LC; SP‐HC‐TS‐TEV‐GFP‐8×HT‐3C‐3×FT‐pFBd
12	GluA1	11B8‐STT	ScFv, SP‐VH‐VL‐STII‐pFBd
13		11B8‐CST	ScFv, SP‐VH‐VL‐FC‐3C‐2×STII‐pFBd
14		11B8‐GFH	Fab, SP‐LC; SP‐VH‐VL‐TS‐mEGFP‐3C‐3×FT‐8×HT‐pFBd
15		11B8‐GHS	Fab, SP‐LC; SP‐VH‐VL‐TS‐GFP‐3C‐2×STII‐pFBd
16		11B8‐CST	Fab, SP‐LC; SP‐HC‐FC‐3C‐2×STII‐pFBd
17	GluA2	15F1‐CSG	Fab, SP‐LC; SP‐HC‐FC‐3C‐2×STII‐TEV‐mEGFP‐pFBd
18		15F1‐mSS	Fab, SP‐LC‐mScarlet‐3C‐SpT; SP‐HC‐FC‐SuT‐8×HT‐AvT‐pFBd
19		15F1‐CTS	Fab, SP‐LC; SP‐HC‐FC‐3C‐2×STII‐pFBd
20		15F1‐SmCF	Fab, SP‐LC‐2×STII; SP‐HC‐TS‐mCherry‐3C‐3×FT‐pFBd
21		15F1‐GST	Fab, SP‐LC; SP‐HC‐TS‐mEGFP‐3C‐2× STII‐pFBd
22		15F1‐mCH	Fab, SP‐LC; SP‐HC‐mCherry‐3C‐3×FT‐TEV‐8×HT‐pFBd
23		15F1‐mCHFv	ScFv, SP‐VH‐VL‐TS‐mCherry‐3C‐3×FT‐TEV‐8×HT‐pFBd
24		15F1‐mCF	ScFv, SP‐VH‐VL‐TS‐mCherry‐3C‐3×FT‐pFBd
25	GluA3	5B2‐HIT	Fab, SP‐LC; SP‐HC‐TS‐8×HT‐pFBd
26		5B2‐mCF	Fab, SP‐LC; SP‐HC‐3C‐mCherry‐2×FT‐pFBd
27		5B2‐mVF	Fab, SP‐LC; SP‐HC‐3C‐mVenus‐2×FT‐pFBd
28	TARP γ‐2	4B2‐GST	Fab, SP‐LC; SP‐HC‐TS‐mEGFP‐3C‐2×STII‐pFBd
29	TARP γ‐8	9B5‐GST	Fab, SP‐LC; SP‐HC‐TS‐mEGFP‐3C‐2×STII‐pFBd
30		15G7‐GST	Fab, SP‐LC; SP‐HC‐TS‐mEGFP‐3C‐2×STII‐pFBd
31		13A8‐GST	Fab, SP‐LC; SP‐HC‐TS‐mEGFP‐3C‐2×STII‐pFBd

^*a*^ All genes were cloned into the pFastBac dual vector, which has an ampicillin resistance marker.

^*b*^ Abbreviations: pFBd – pFastBac vector, SP – Signal peptide, LC – light chain, HC – Heavy chain, TS– Thrombin protease recognition site, STII – Strep tag II, FC – Free cysteine for Cys‐reactive reagent coupling, HT – His affinity tag, 3C – HRV 3C protease recognition site, FT – FLAG affinity tag, SpT – Spy affinity tag, SuT – Sumo protease recognition tag, AvT – Avi tag for protein biotinylation, TEV – TEV protease recognition site, L – Linker. mEGFP, mCherry, mScarlet, and mVenus denote the fluorescent proteins.

^*c*^ 2B5 Fab (specific to GluN2B) sequences were retrieved from a public repository (Tajima et al., 2022).

### Time Considerations

The preparation of PLs is a multi‐step process completed over 4 days. Liposome preparation (Basic Protocol 1, steps 1 to 4) is carried out on the first day, requiring ∼2 hr, followed by overnight evaporation in a desiccator. The next day, the dried lipid film is resuspended and subjected to extrusion (Basic Protocol 1, steps 5 to 10) to achieve uniform liposome size. On the third day, antigen is reconstituted into the liposomes (Basic Protocol 1, steps 11 to 13), a process taking ∼3 hr. Finally, the efficiency of antigen incorporation is assessed using SDS‐PAGE and FSEC on the fourth day (Basic Protocol 1, steps 14 to 16), which requires around 3 to 5 hr.

Immunizations in mice (Basic Protocol 1, steps 17 and 18) are performed twice, on day 5 and day 19, each taking ∼30 min. Following immunization, blood collection and serum titer analysis (Basic Protocol 1, steps 19 and 20) are conducted on day 31, requiring 4 hr. Serum screening using FSEC (Basic Protocol 1, steps 21 to 23) takes 6 hr. On day 51, spleen injections (Basic Protocol 1, steps 24 to 38) are carried out under sterile conditions for ∼2 hr with surgical expertise. Myeloma cell growth (Basic Protocol 1, steps 39 to 43) is monitored from day 46 to day 52.

Hybridoma fusion using PEG (Basic Protocol 1, steps 44 to 67) is performed on day 53 to generate hybrid clones. After a 12‐day incubation period for hybridoma selection and expansion, fusion screening is conducted on day 66 using an ELISA (Basic Protocol 1, steps 68 and 69 and Support Protocol 1), which takes ∼8 hr. This is followed by native and denaturing ELISA (Basic Protocol 1, steps 70 and 71 and Support Protocol 1) to evaluate conformation‐specific antibodies and subunit‐specificity ELISA (Basic Protocol 1, step 72 and Support Protocol 1) to assess target selectivity, each lasting ∼6 hr. Candidate hybridomas are subsequently subcultured (Basic Protocol 1, step 73) on the same day, over 6 hr, for further propagation and downstream characterization. The duration of further hybridoma characterization and the production of mAbs and Fabs (Basic Protocol 1, steps 74 to 86; Basic Protocol 2; and Support Protocol 2) is dependent on the scale of cell expansion and the efficiency of antibody expression, varying according to experimental requirements.

### Author Contributions

**Natalie** Sheldon: Formal analysis; investigation; writing ‐ original draft; writing ‐ review and editing. **Gunasekaran** Dhandapani: Formal analysis; investigation; writing ‐ original draft; writing ‐ review and editing. **Junhoe** Kim: Formal analysis; investigation; writing ‐ original draft; writing ‐ review and editing. **Cathy J**. Spangler: Formal analysis; investigation. **Chengli** Fang: Formal analysis; investigation. **Jumi** Park: Formal analysis; investigation. **Prashant** Rao: Formal analysis; investigation. **Eric** Gouaux: Conceptualization; formal analysis; funding acquisition; investigation; project administration; supervision; writing ‐ original draft; writing ‐ review and editing.

### Conflict of Interest

The authors declare no conflict of interest.

## Supporting information


**Figure S1** Assessment of specificity, cross‐reactivity, and competitive binding of antibodies specific to iGluRs.
**Figure S2** FSEC screening for cross‐reactivity with varying antibody ratios.
**Figure S3** ForteBio Octet RED384 binding affinity determination.
**Table S1** List of potential iGluR gene candidates cloned into the pEG BacMam vector for heterologous expression.
**Table S2** Antibody sequences for the heavy (VH‐CH) and light (VL‐CL) chains of in‐house‐generated antibodies targeting iGluR and its auxiliary protein.

## Data Availability

Data sharing is not applicable to this article as no datasets were generated or analyzed during the current study.

## References

[cpz170142-bib-0001] Arlotta, K. J. , & Owen, S. C. (2019). Antibody and antibody derivatives as cancer therapeutics. WIREs Nanomedicine and Nanobiotechnology, 11(5), e1556. 10.1002/wnan.1556 30968595

[cpz170142-bib-0002] Bissen, D. , Foss, F. , & Acker‐Palmer, A. (2019). AMPA receptors and their minions: Auxiliary proteins in AMPA receptor trafficking. Cellular and Molecular Life Sciences, 76(11), 2133–2169. 10.1007/s00018-019-03068-7 30937469 PMC6502786

[cpz170142-bib-0003] Bloch, J. S. , Mukherjee, S. , Kowal, J. , Filippova, E. V. , Niederer, M. , Pardon, E. , Steyaert, J. , Kossiakoff, A. A. , & Locher, K. P. (2021). Development of a universal nanobody‐binding Fab module for fiducial‐assisted cryo‐EM studies of membrane proteins. Proceedings of the National Academy of Sciences, 118(47), e2115435118. 10.1073/pnas.2115435118 PMC8617411 34782475

[cpz170142-bib-0004] Carolin, B. , Anne‐Sophie, H. , Thomas, S. , Maximilian, T. S. , Sebastian, M. , Ulrike, E. , Ralf, J. , Erin, M. S. , & Mike, H. (2019). Super‐resolution imaging and estimation of protein copy numbers at single synapses with DNA‐point accumulation for imaging in nanoscale topography. Neurophotonics, 6(3), 035008. 10.1117/1.NPh.6.3.035008 31637284 PMC6795074

[cpz170142-bib-0005] Chen, C. L. , Klose, T. , Sun, C. , Kim, A. S. , Buda, G. , Rossmann, M. G. , Diamond, M. S. , Klimstra, W. B. , & Kuhn, R. J. (2022). Cryo‐EM structures of alphavirus conformational intermediates in low pH‐triggered prefusion states. Proceedings of the National Academy of Sciences, 119(30), e2114119119. 10.1073/pnas.2114119119 PMC9335222 35867819

[cpz170142-bib-0006] Choe, H.‐W. , Kim, Y. J. , Park, J. H. , Morizumi, T. , Pai, E. F. , Krauß, N. , Hofmann, K. P. , Scheerer, P. , & Ernst, O. P. (2011). Crystal structure of metarhodopsin II. Nature, 471(7340), 651–655. 10.1038/nature09789 21389988

[cpz170142-bib-0007] Chou, T. H. , Tajima, N. , Romero‐Hernandez, A. , & Furukawa, H. (2020). Structural basis of functional transitions in mammalian NMDA receptors. Cell, 182(2), 357–371. e313. 10.1016/j.cell.2020.05.052 32610085 PMC8278726

[cpz170142-bib-0008] Galfre, G. , Howe, S. C. , Milstein, C. , Butcher, G. W. , & Howard, J. C. (1977). Antibodies to major histocompatibility antigens produced by hybrid cell lines. Nature, 266(5602), 550–552. 10.1038/266550a0 558524

[cpz170142-bib-0009] Gallagher, S. R. (2012). One‐dimensional SDS gel electrophoresis of proteins. Current Protocols in Protein Science, 68(1), 10.11.11–10.11.44. 10.1002/0471140864.ps1001s68 22470126

[cpz170142-bib-0010] Gearing, A. J. H. , Thorpe, R. , Spitz, L. , & Spitz, M. (1985). Use of ‘single shot’ intrasplenic immunization for production of monoclonal antibodies specific for human IgM. Journal of Immunological Methods, 76(2), 337–343. 10.1016/0022-1759(85)90311-4 3919104

[cpz170142-bib-0011] Giannone, G. , Hosy, E. , Levet, F. , Constals, A. , Schulze, K. , Sobolevsky, A. I. , Rosconi, M. P. , Gouaux, E. , Tampé, R. , Choquet, D. , & Cognet, L. (2010). Dynamic superresolution imaging of endogenous proteins on living cells at ultra‐high density. Biophysical Journal, 99(4), 1303–1310. 10.1016/j.bpj.2010.06.005 20713016 PMC2920718

[cpz170142-bib-0012] Goehring, A. , Lee, C.‐H. , Wang, K. H. , Michel, J. C. , Claxton, D. P. , Baconguis, I. , Althoff, T. , Fischer, S. , Garcia, K. C. , & Gouaux, E. (2014). Screening and large‐scale expression of membrane proteins in mammalian cells for structural studies. Nature Protocols, 9(11), 2574–2585. 10.1038/nprot.2014.173 25299155 PMC4291175

[cpz170142-bib-0013] Gouaux, E. (2004). Structure and function of AMPA receptors. The Journal of Physiology, 554(Pt 2), 249–253. 10.1113/jphysiol.2003.054320 14645452 PMC1664757

[cpz170142-bib-0014] Grisshammer, R. (2017). New approaches towards the understanding of integral membrane proteins: A structural perspective on G protein‐coupled receptors. Protein Science, 26(8), 1493–1504. 10.1002/pro.3200 28547763 PMC5521582

[cpz170142-bib-0015] Gui, M. , Song, W. , Zhou, H. , Xu, J. , Chen, S. , Xiang, Y. , & Wang, X. (2017). Cryo‐electron microscopy structures of the SARS‐CoV spike glycoprotein reveal a prerequisite conformational state for receptor binding. Cell Research, 27(1), 119–129. 10.1038/cr.2016.152 28008928 PMC5223232

[cpz170142-bib-0016] Hattori, M. , Hibbs, R. E. , & Gouaux, E. (2012). A fluorescence‐detection size‐exclusion chromatography‐based thermostability assay for membrane protein precrystallization Screening. Structure, 20(8), 1293–1299. 10.1016/j.str.2012.06.009 22884106 PMC3441139

[cpz170142-bib-0017] Hibbs, R. E. , & Gouaux, E. (2011). Principles of activation and permeation in an anion‐selective Cys‐loop receptor. Nature, 474(7349), 54–60. 10.1038/nature10139 21572436 PMC3160419

[cpz170142-bib-0018] Hosokawa, T. , Liu, P.‐W. , Cai, Q. , Ferreira, J. S. , Levet, F. , Butler, C. , Sibarita, J.‐B. , Choquet, D. , Groc, L. , Hosy, E. , Zhang, M. , & Hayashi, Y. (2021). CaMKII activation persistently segregates postsynaptic proteins via liquid phase separation. Nature Neuroscience, 24(6), 777–785. 10.1038/s41593-021-00843-3 33927400

[cpz170142-bib-0019] Huston, J. S. , Levinson, D. , Mudgett‐Hunter, M. , Tai, M. S. , Novotný, J. , Margolies, M. N. , Ridge, R. J. , Bruccoleri, R. E. , Haber, E. , & Crea, R. (1988). Protein engineering of antibody binding sites: Recovery of specific activity in an anti‐digoxin single‐chain Fv analogue produced in Escherichia coli. Proceedings of the National Academy of Sciences, 85(16), 5879–5883. 10.1073/pnas.85.16.5879 PMC281868 3045807

[cpz170142-bib-0020] Jin, S. , Sun, Y. , Liang, X. , Gu, X. , Ning, J. , Xu, Y. , Chen, S. , & Pan, L. (2022). Emerging new therapeutic antibody derivatives for cancer treatment. Signal Transduction and Targeted Therapy, 7(1), 39. 10.1038/s41392-021-00868-x 35132063 PMC8821599

[cpz170142-bib-0021] Kandari, D. , & Bhatnagar, R. (2023). Antibody engineering and its therapeutic applications. International Reviews of Immunology, 42(2), 156–183. 10.1080/08830185.2021.1960986 34355613

[cpz170142-bib-0022] Kawate, T. , & Gouaux, E. (2006). Fluorescence‐detection size‐exclusion chromatography for precrystallization screening of integral membrane proteins. Structure, 14(4), 673–681. 10.1016/j.str.2006.01.013 16615909

[cpz170142-bib-0023] Krishnamurthy, H. , & Gouaux, E. (2012). X‐ray structures of LeuT in substrate‐free outward‐open and apo inward‐open states. Nature, 481(7382), 469–474. 10.1038/nature10737 22230955 PMC3306218

[cpz170142-bib-0024] Maeda, S. , Koehl, A. , Matile, H. , Hu, H. , Hilger, D. , Schertler, G. F. X. , Manglik, A. , Skiniotis, G. , Dawson, R. J. P. , & Kobilka, B. K. (2018). Development of an antibody fragment that stabilizes GPCR/G‐protein complexes. Nature Communications, 9(1), 3712. 10.1038/s41467-018-06002-w PMC6137068 30213947

[cpz170142-bib-0025] Matsui, A. , Spangler, C. , Elferich, J. , Shiozaki, M. , Jean, N. , Zhao, X. , Qin, M. , Zhong, H. , Yu, Z. , & Gouaux, E. (2024). Cryo‐electron tomographic investigation of native hippocampal glutamatergic synapses. eLife, 13, RP98458. 10.7554/eLife.98458 39495821 PMC11534335

[cpz170142-bib-0026] Nguyen, T. , & Song, J. (2021). Direct IgG epitope mapping on bacterial AB toxins by cryo‐EM. STAR Protocols, 2(4), 100852. 10.1016/j.xpro.2021.100852 34647035 PMC8496303

[cpz170142-bib-0027] Ove Nilsson, B. , & Larsson, A. (1990). Intrasplenic immunization with minute amounts of antigen. Immunology Today, 11, 10–12. 10.1016/0167-5699(90)90004-S 2405872

[cpz170142-bib-0028] Pedrioli, A. , & Oxenius, A. (2021). Single B cell technologies for monoclonal antibody discovery. Trends in Immunology, 42(12), 1143–1158. 10.1016/j.it.2021.10.008 34743921

[cpz170142-bib-0029] Penmatsa, A. , Wang, K. H. , & Gouaux, E. (2013). X‐ray structure of dopamine transporter elucidates antidepressant mechanism. Nature, 503(7474), 85–90. 10.1038/nature12533 24037379 PMC3904663

[cpz170142-bib-0030] Penmatsa, A. , Wang, K. H. , & Gouaux, E. (2015). X‐ray structures of Drosophila dopamine transporter in complex with nisoxetine and reboxetine. Nature Structural & Molecular Biology, 22(6), 506–508. 10.1038/nsmb.3029 PMC4608549 25961798

[cpz170142-bib-0031] Rao, P. , & Gouaux, E. (2023). Purification and biochemical analysis of native AMPA receptors from three different mammalian species. PLoS ONE, 18(3), e0275351. 10.1371/journal.pone.0275351 36930594 PMC10022779

[cpz170142-bib-0032] Rathnayake, S. S. , Erramilli, S. K. , Kossiakoff, A. A. , & Vecchio, A. J. (2024). Cryo‐EM structures of Clostridium perfringens enterotoxin bound to its human receptor, claudin‐4. Structure, 32(11), 1936–1951. e1935. 10.1016/j.str.2024.09.015 39383874 PMC11560561

[cpz170142-bib-0033] Refaat, A. , Yap, M. L. , Pietersz, G. , Walsh, A. P. G. , Zeller, J. , del Rosal, B. , Wang, X. , & Peter, K. (2022). In vivo fluorescence imaging: Success in preclinical imaging paves the way for clinical applications. Journal of Nanobiotechnology, 20(1), 450. 10.1186/s12951-022-01648-7 36243718 PMC9571426

[cpz170142-bib-0034] Robertson, M. J. , Papasergi‐Scott, M. M. , He, F. , Seven, A. B. , Meyerowitz, J. G. , Panova, O. , Peroto, M. C. , Che, T. , & Skiniotis, G. (2022). Structure determination of inactive‐state GPCRs with a universal nanobody. Nature Structural & Molecular Biology, 29(12), 1188–1195. 10.1038/s41594-022-00859-8 PMC12014012 36396979

[cpz170142-bib-0035] Selvakumar, P. , Fernández‐Mariño, A. I. , Khanra, N. , He, C. , Paquette, A. J. , Wang, B. , Huang, R. , Smider, V. V. , Rice, W. J. , Swartz, K. J. , & Meyerson, J. R. (2022). Structures of the T cell potassium channel Kv1.3 with immunoglobulin modulators. Nature Communications, 13(1), 3854. 10.1038/s41467-022-31285-5 PMC9253088 35788586

[cpz170142-bib-0036] Shaffer, P. L. , Goehring, A. , Shankaranarayanan, A. , & Gouaux, E. (2009). Structure and mechanism of a Na^+^‐independent amino acid transporter. Science, 325(5943), 1010–1014. 10.1126/science.1176088 19608859 PMC2851542

[cpz170142-bib-0037] Stroebel, D. , & Paoletti, P. (2021). Architecture and function of NMDA receptors: An evolutionary perspective. The Journal of Physiology, 599(10), 2615–2638. 10.1113/JP279028 32786006

[cpz170142-bib-0038] Tajima, N. , Simorowski, N. , Yovanno, R. A. , Regan, M. C. , Michalski, K. , Gómez, R. , Lau, A. Y. , & Furukawa, H. (2022). Development and characterization of functional antibodies targeting NMDA receptors. Nature Communications, 13(1), 923. 10.1038/s41467-022-28559-3 PMC8854693 35177668

[cpz170142-bib-0039] Traynelis, S. F. , Wollmuth, L. P. , McBain, C. J. , Menniti, F. S. , Vance, K. M. , Ogden, K. K. , Hansen, K. B. , Yuan, H. , Myers, S. J. , & Dingledine, R. (2010). Glutamate receptor ion channels: Structure, regulation, and function. Pharmacological Reviews, 62(3), 405–496. 10.1124/pr.109.002451 20716669 PMC2964903

[cpz170142-bib-0040] Wang, K. H. , Penmatsa, A. , & Gouaux, E. (2015). Neurotransmitter and psychostimulant recognition by the dopamine transporter. Nature, 521(7552), 322–327. 10.1038/nature14431 25970245 PMC4469479

[cpz170142-bib-0041] Wang, Y. , Zhuang, Y. , DiBerto, J. F. , Zhou, X. E. , Schmitz, G. P. , Yuan, Q. , Jain, M. K. , Liu, W. , Melcher, K. , Jiang, Y. , Roth, B. L. , & Xu, H. E. (2023). Structures of the entire human opioid receptor family. Cell, 186(2), 413–427. e417. 10.1016/j.cell.2022.12.026 36638794

[cpz170142-bib-0042] Warke, A. , & Momany, C. (2007). Addressing the protein crystallization bottleneck by cocrystallization. Crystal Growth & Design, 7(11), 2219–2225. 10.1021/cg700702c

[cpz170142-bib-0043] Wu, K. L. , Yu, C. , Lee, C. , Zuo, C. , Ball, Z. T. , & Xiao, H. (2021). Precision modification of native antibodies. Bioconjugate Chemistry, 32(9), 1947–1959. 10.1021/acs.bioconjchem.1c00342 34428033 PMC8744088

[cpz170142-bib-0044] Yang, D. , Singh, A. , Wu, H. , & Kroe‐Barrett, R. (2017). Determination of high‐affinity antibody‐antigen binding kinetics using four biosensor platforms. Journal of Visualized Experiments: JoVE, (122), 10.3791/55659 PMC5564993 28448040

[cpz170142-bib-0045] Youn, Y. , Lau, G. W. , Lee, Y. , Maity, B. K. , Gouaux, E. , Chung, H. J. , & Selvin, P. R. (2023). Quantitative DNA‐PAINT imaging of AMPA receptors in live neurons. Cell Reports Methods, 3(2), 100408. 10.1016/j.crmeth.2023.100408 36936077 PMC10014303

[cpz170142-bib-0046] Yu, J. , Rao, P. , Clark, S. , Mitra, J. , Ha, T. , & Gouaux, E. (2021). Hippocampal AMPA receptor assemblies and mechanism of allosteric inhibition. Nature, 594(7863), 448–453. 10.1038/s41586-021-03540-0 33981040 PMC8270219

[cpz170142-bib-0047] Zhao, Y. , Chen, S. , Swensen, A. C. , Qian, W. J. , & Gouaux, E. (2019). Architecture and subunit arrangement of native AMPA receptors elucidated by cryo‐EM. Science, 364(6438), 355–362. 10.1126/science.aaw8250 30975770 PMC6701862

