# Supplementary material for: Generation of Conformation‐Specific Monoclonal Antibodies for Integral Membrane Proteins
Source: Curr Protoc. 2025 May 26;5(5):e70142. doi: 10.1002/cpz1.70142 (PMC12105683; doi:10.1002/cpz1.70142)
Supplement: Supplementary file 1 — Figure S1 Assessment of specificity, cross‐reactivity, and competitive binding of antibodies specific to iGluRs. Figure S2 FSEC screening for cross‐reactivity with varying antibody ratios. Figure S3 ForteBio Octet RED384 binding affinity determination. Table S1 List of potential iGluR gene candidates cloned into the pEG BacMam vector for heterologous expression. Table S2 Antibody sequences for the heavy (VH‐CH) and light (VL‐CL) chains of in‐house‐generated antibodies targeting iGluR and its auxiliary protein. [file CPZ1-5-0-s001.pdf]

Supplementary Information for

Generation of conformation-specific monoclonal antibodies for integral membrane proteins

Natalie Sheldon[1,2,^], Gunasekaran Dhandapani[1,^], Junhoe Kim[1], Cathy J Spangler[1], Chengli Fang[1], Jumi Park[1], Prashant Rao[1,3], Eric Gouaux[1,2*]

[1]Vollum Institute, Oregon Health and Science University, 3232 SW Research Drive, Portland, OR 97239
[2]Howard Hughes Medical Institute, Oregon Health and Science University, 3232 SW Research Drive, Portland, OR 97239
[3]Present address: Calico Life Sciences LLC, 1170 Veterans Blvd, South San Francisco, CA, 94080

*Correspondence to: Eric Gouaux, gouauxe@ohsu.edu, 503-494-5535.

^These authors contributed equally to this work.

Table of Contents:

Fig. S1
Fig. S2
Fig. S3
Table S1
Table S2

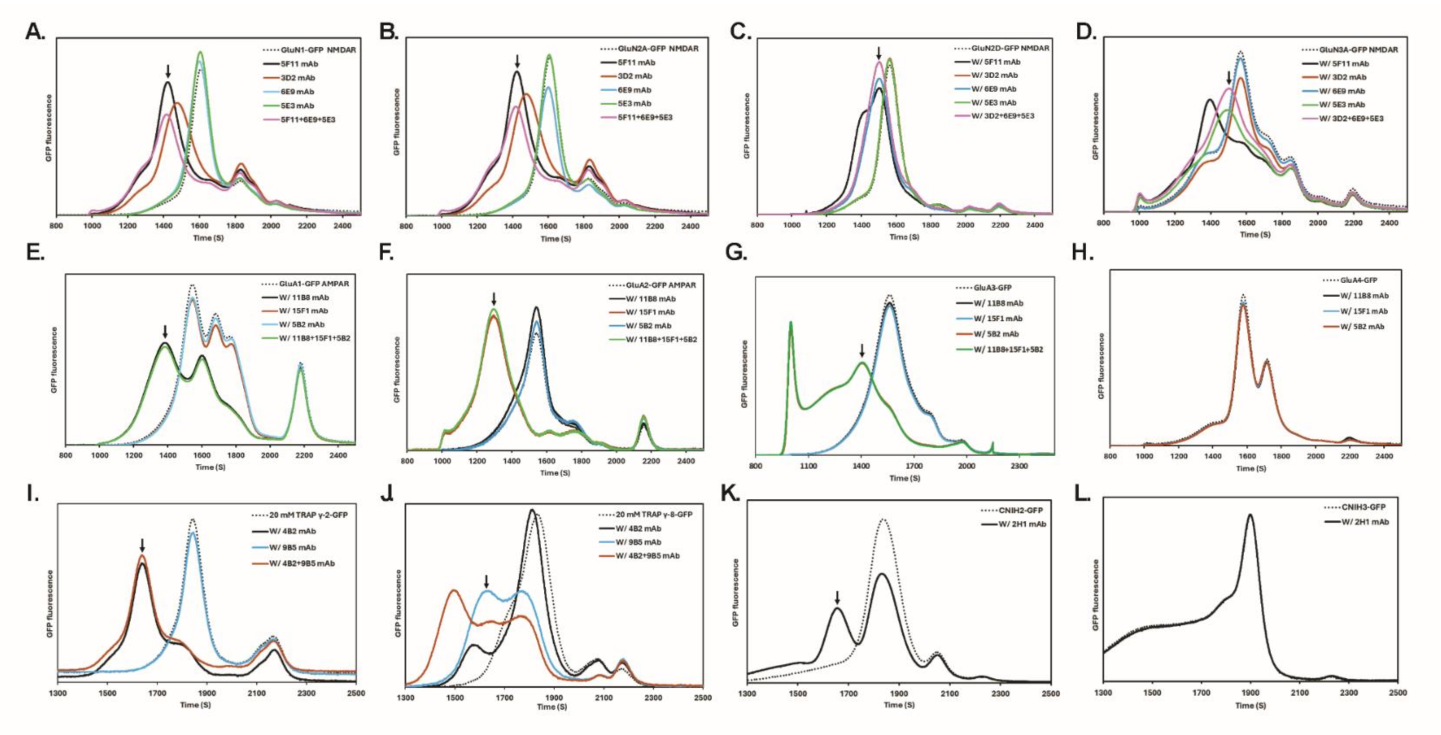

Figure S1. Assessment of specificity, cross-reactivity, and competitive binding of antibodies specific to iGluRs. The FSEC profiles shown in panels A-D represent the NMDAR heterotetrameric receptor subunits: A) GluN1-GFP, B) GluN2A-GFP, C) GluN2D-GFP, and D) GluN3A-GFP. Panels E-H display the homomeric GluA1-A4 subunit tagged with GFP, while I-J show TARP γ-2-GFP and TARP γ-8-GFP, respectively. Panels K-L illustrate the CNIH2-GFP and CNIH3-GFP subunits. The receptor-mAb complexes (1:2 ratio) were detected by GFP fluorescence (excitation/emission at 480/510 nm), using a Superose 6 10/300 GL column with a flow rate of 0.5 ml/min. Antibody-bound GluRs (indicated by arrows) elute significantly earlier than un-complexed receptors.

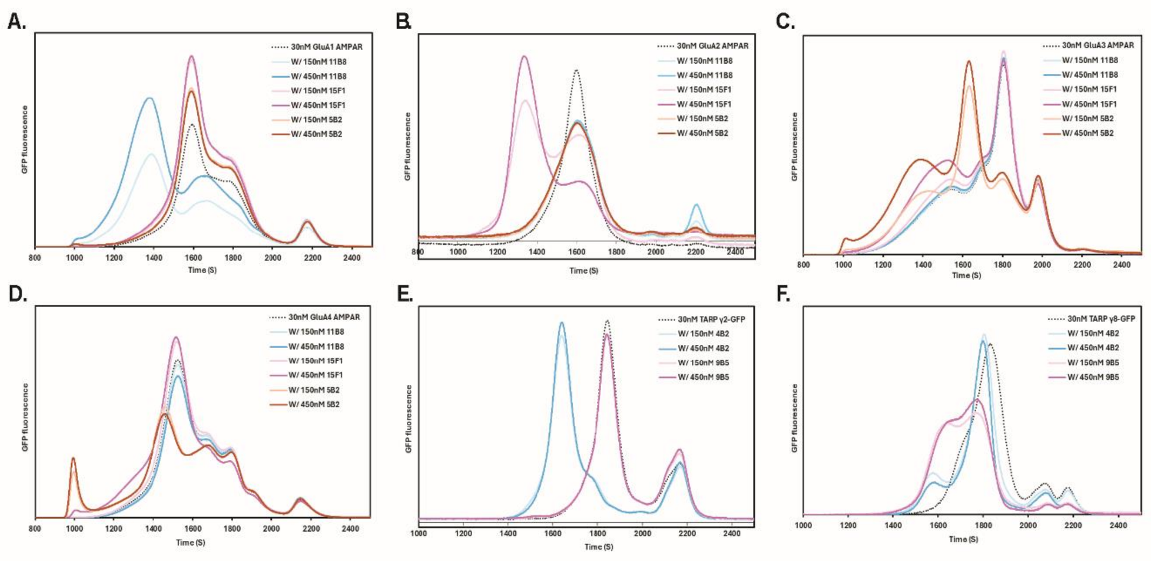

Figure S2. FSEC screening for cross-reactivity with varying antibody ratios. A) FSEC traces of GFP-tagged GluA1 AMPARs incubated with 1:5 and 1:15 ratios of 11B8, 15F1, and 5B2 mAbs (excitation/emission at 480/510 nm). Black dotted lines represent unbound receptors, while colored lines indicate receptors bound to antibodies. B) FSEC traces of GluA2 AMPARs. C) FSEC traces of GluA3 AMPARs. D) FSEC traces of GluA4 AMPARs. E-F) FSEC traces of TARP γ-2 and γ-8, respectively.

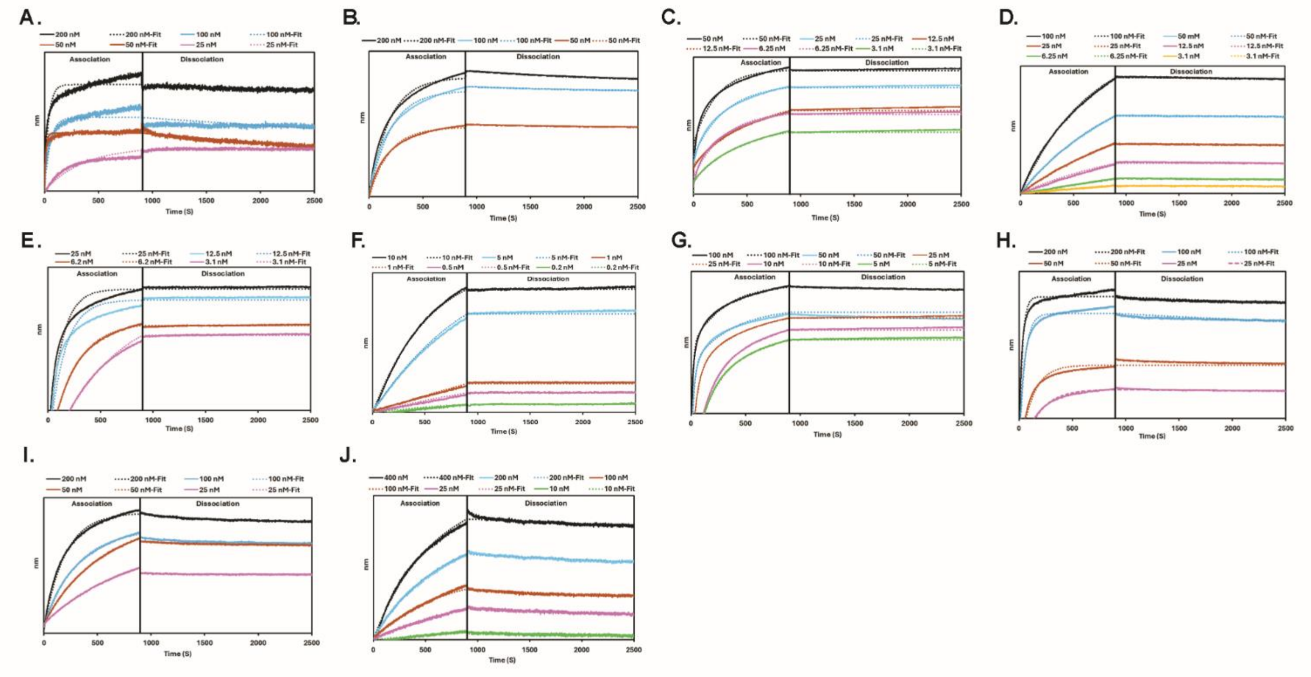

Figure S3. ForteBio - Octet® RED384 binding affinity determination. A-D) Octet kinetic sensorgrams showing the binding of GluN1-5F11 mAb (A), GluN2A-3D2 mAb (B), GluN2D-6E9 mAb (C), and GluN3A-5E3 mAb complexes. Anti-Mouse IgG Fc Capture (AMC) biosensors were used to capture the mAbs, followed by the addition of varying concentrations of receptor. A 0.075% LMNG in TBS buffer was used as a negative control. The analytes are represented by different colors, reflecting repeated dilutions of rGluR protein from higher to lower concentrations. The binding affinity (KD) was calculated and is presented in Table 2. The overlaid colored dotted lines show kinetic fitting of the binding response signals to a 1:1 interaction model for each GluR concentration. E-G) Binding of 11B8, 15F1, and 5B2 mAbs to GluA1-A3 AMPARs. H-I) Binding of mAb 4B2 to TARP γ-2 and mAb 9B5 to TARP γ-8. J) Binding of mAb 2H1 to CNIH2.

Table S1. The list of potential iGluR gene candidates cloned into the pEG-BacMam vector for heterologous expression is provided.

S. No	Antigen	Construct information	Species
1	GluN1	Uniprot: A0A1L8F5J9, GluN1/GluN2B NMDAR, truncated after residue 836, and reconstituted in PL	Xenopus laevis
2	GluN2A	Uniprot: G3V9C5, GluN1/GluN2A NMDAR, truncated after residue 866, and reconstituted in PL	Rattus norvegicus
3	GluN2D	Uniprot: Q62645, GluN1/GluN2D NMDAR, truncated after residue 889, and reconstituted in PL	Rattus norvegicus
4	GluN3A	Uniprot: Q9R1M7, GluN1/GluN3A full-length subunit, truncated after residue 970, and reconstituted in PL	Rattus norvegicus
5	GluA1	Uniprot code: P19490, GluA1 AMPAR flip variant, unedited (Q), truncated after residue 840, C-terminal Strep tag II, and reconstituted in PL	Rattus norvegicus
6	GluA2	Uniprot: P19491, GluA2 AMPAR flop variant, contains point mutations R586Q, and reconstituted in PL	Rattus norvegicus
7	GluA3	Uniprot code: P19492, GluA3 AMPAR flop variant, edited (R), truncated after residue 852, C terminal Strep tag II, and reconstituted in PL	Rattus norvegicus
8	TARP γ-2	Uniport: Q71RJ2, full length protein, and reconstituted in PL	Mus musculus
9	TARP γ-8	Uniport: Q8VHW2, full length protein, and reconstituted in PL	Mus musculus

10	Cornichon-2	Uniprot: Q5BJU5 full length, C terminal Strep tag II, and reconstituted in PL	Rattus norvegicus

Table S2. The antibody sequences for the heavy (VH-CH) and light (VL-CL) chains of in-house generated antibodies targeting iGluR and its auxiliary protein were provided.

5F11 Fab	SP-VH-CH<br><br>MVSAIVLYVLLAAAAHSAFAQGQMQQSGAELVKPGASVKLSCKTSGFSFSTSYINWLKQKPGQSLEWIAWIYAGTDGTSYN QKFTDKAQLTVDTSSSTAYMQFSSLTTDDSAIYYCAREGIYYGNYAGFTYWGQGTLVTVSAAKTTPPSVYPLAPGSAAQTN SMVTLGCLVKGYFPEPVTVTWNSGSLSSGVHTFPAVLQSDLYTLSSSVTVPSSTWPSETVTCNVAHPASSTKVDKK-<br><br>SP-VL-CL<br><br>MVSAIVLYVLLAAAAHSAFADIVMTQSPSSLAVTAGEKVTMRCKSSQSLLWSVNQNNYLSWYQQKQGQPPKLLIYGASIRES WVPDRFTGSGSGTDFTLTISNVHAEDLAVYYCQHNHGSFLPYTFGGGTKLEIKRADAAPTVSIFPPSSEQLTSGGASVVCFL NNFYPKDINVKWKIDGSERQNGVLNSWTDQDSKDSTYSMSSTLTLTKDEYERHNSYTCEATHKTSTSPIVKSFNRNECSN-
3D2 Fab	SP-VH-CH<br><br>MVSAIVLYVLLAAAAHSAFAQVQLQQSGAELMKPGASVKISCKATGYTFRSYWIEWLKQRPGHGLEWIGEILPGSGRTNYNE KFKGKATITADTSSNTAYMQLSSLTSEDSAVYYCTRSRGTMITREFTYWGQGALVTVSAAAKTTPPSVYPLAPGSAAQTNS MVTLGCLVKGYFPEPVTVTWNSGSLSSGVHTFPAVLQSDLYTLSSSVTVPSSTWPSETVTCNVAHPASSTKVDKK-<br><br>SP-VL-CL<br><br>MVSAIVLYVLLAAAAHSAFADIVLTQSPASLAVSLGQRATISCRASESVEYYGTTLMQWYQQKPGQPPKLLIYAASNVDSGVP ARFSGSGSGTDFSLNIHPVEEDDIAMYFCQQSRKVPSTFGGGTKLEIKRADAAPTVSIFPPSSEQLTSGGASVVCFLNNFYP KDINVKWKIDGSERQNGVLNSWTDQDSKDSTYSMSSTLTLTKDEYERHNSYTCEATHKTSTSPIVKSFNRNECSN-

5E3<br>Fab	SP-VH-CH<br><br>MVSAIVLYVLLAAAAHSAFAQVQLQQPGAELVRPGASVKLSCKASGYSFTSYWMNWVKQRPGQGLEWIGMIHPSDSESRL<br>NQKFKDKATLTVDKSSSTAYMQLRSPTSEDSAVYYCARSAYYRSFDYWGQGTTLTVSSAAKTTPPSVYPLAPGSAASTNS<br>MVTLGCLVKGYFPEPVTVTWNSGSLSSGVHTFPAVLQSDLYTLSSSVTVPSSTWPSETVTCNVAHPASSTKVDKK-<br><br>SP-VL-CL<br><br>MVSAIVLYVLLAAAAHSAFADVLMTQTPLSLPVSLGDQASISCRSSQNIVHSNGNTYLEWYLQKPGQSPKLLIHKVSNRFSGV<br>PDRFSGSGSGTDFTLKISRVEAEDLGVYYCFQGSHVPYTFGGGTKLEIKRADAAPTVSIFPPSSEQLTSGGASVVCFLNNFY<br>PKDINVKWKIDGSERQNGVLNSWTDQDSKDSTYSMSSTLTLTKDEYERHNSYTCEATHKTSTSPIVKSFNRNECSN-
3G9<br>Fab	SP-VH-CH<br><br>MVSAIVLYVLLAAAAHSAFAKVQLQQSGAGLVKPGASVKLSCKASGYTFTDYIIHWIKQRSGQGLEWIGWFYPGSGSIKYNE<br>KFKDKATLTADYSSSTVYMELSRLTSEDSAVYFCARHEHRGYYGDYPMDYWGQGTSVTVSSAAKTTPPSVYPLAPGSAAS<br>TNSMVTLGCLVKGYFPEPVTVTWNSGSLSSGVHTFPAVLQSDLYTLSSSVTVPSSTWPSETVTCNVAHPASSTKVDKK-<br><br>SP-VL-CL<br><br>MVSAIVLYVLLAAAAHSAFADIVMSQSPSSLAVSVGEKVTMSCKSSQSLLYSNNQKNYLAWYQQKPGQSPKLLIYWASTRE<br>SGVPDRFTGSGSGTDFTLTISSVKAEDLAVYYCQQYYSYPPYTFGGGTKLEIKRADAAPTVSIFPPSSEQLTSGGASVVCFL<br>NNFYPKDINVKWKIDGSERQNGVLNSWTDQDSKDSTYSMSSTLTLTKDEYERHNSYTCEATHKTSTSPIVKSFNRNECSN-
2D3<br>Fab	SP-VH-CH<br><br>MVSAIVLYVLLAAAAHSAFAEVQLVESGGGLVKPGGSLKLSCAASGFAFSTYDMSWVRQTPEKRLEWVAYISGGGGTTYYP<br>GTVKGRFTISRDNAKNTLYLQMSSLKSEDTAMYYCARQGYGYSMDYWGQGTSVTVSSAAKTTPPSVYPLAPGSAASTNSM<br>VTLGCLVKGYFPEPVTVTWNSGSLSSGVHTFPAVLQSDLYTLSSSVTVPSSTWPSETVTCNVAHPASSTKVDKK-<br><br>SP-VL-CL

	MVSAIVLYVLLAAAAHSAFADVVMTQTPLSLPVSLGDQASISCRSSQSLVHSNGDTYLLWYLQKPGQSPKLLIYKVSNRFSG<br>VPDRFSGSGSGTDFTLKISRVEAEDLGVYFCSQSTHVPRTFGGGTKLEIKRADAAPTVSIFPPSSEQLTSGGASVVCFLNNF<br>YPKDINVKWKIDGSERQNGVLNSWTDQDSKDSTYSMSSTLTLTKDEYERHNSYTCEATHKTSTSPIVKSFNRNECSN-
11B8<br>Fab	SP-VH-CH<br><br>MVSAIVLYVLLAAAAHSAFAMEVKLLESGGGLVQPGGSLKLSCAASGFDFSEYWMSWVRQAPGKGLEWIGEINPDSSSIDY<br>TPSLKDKIIISRDNAKKTLYLQLSKVRSEDTALYYCARPRGNYVVMDYWGQGTSVTVSSAKTTPPSVYPLAPGCGDTTGSSV<br>TLGCLVKGYFPESVTVTWNSGSLSSSVHTFPALLQSGLYTMSSSVTVPSSTWPSQTVTCSVAHPASSTTVDKK-<br><br>SP-VL-CL<br><br>MVSAIVLYVLLAAAAHSAFAMNIVLTQSPASLAVSLGQRATISCRASESVDSYGSSFVHWYQQKPGQPPKLLIFLASKLESGV<br>PARFSGSGSRTDFTLTIDPVEADDAATYYCQQTNEDPYTFGGGTKLEIKRADAAPTVSIFPPSSEQLTSGGASVVCFLNNFY<br>PKDINVKWKIDGSERQNGVLNSWTDQDSKDSTYSMSSTLTLTKDEYERHNSYTCEATHKTSTSPIVKSFNRNEC-
15F1<br>Fab	SP-VH-CH<br><br>MVSAIVLYVLLAAAAHSAFAEIQMTQTTSSLSASLGDRVTISCRASQDISNYLSWYQQKPDGTVKLLIYYTSRLHSGVPSRFS<br>GSGSGIDYSLTINNLEQEDFATYFCQQGNTLPLTFGAGTKLELKRADAAPTVSIFPPSSEQLTSGGASVVCFLNNFYPKDINV<br>KWKIDGSERQNGVLNSWTDQDSKDSTYSMSSTLTLTKDEYERHNSYTCEATHKTSTSPIVKSFNRNECSN-<br><br>SP-VL-CL<br><br>MVSAIVLYVLLAAAAHSAFAQAQLKESGPGLVAPSQSLSITCTVSGFSLTNYGVHWVRQPPGKGLEWLGVIWAGGSTNYNS<br>ALMSRVSISKDNSKSQVFLKMNSLQTDDTVMYYCAREDYDYDWHFDVWGAGTTVTVSSAKTTPPSVYPLAPGSAAQTNSM<br>VTLGCLVKGYFPEPVTVTWNSGSLSSGVHTFPAVLQSDLYTLSSSVTVPSSTWPSETVTCNVAHPASSTKVDKKIVPRDAG<br>AKPC-
5B2<br>Fab	SP-VH-CH<br><br>MVSAIVLYVLLAAAAHSAFAMEVQLQESGPSLVKPSQTLSLTCSVTGDSITSGYWNWIRKFPGNKLEYMGHISYRGSTYYNP<br>SLKSRISITRDTSKNQYYLQLNSVTTEDTATYYCASRSYDEGFDSWGQGTTLTVSSAKTTPPSVYPLAPGSAAQTNSMVTLG<br>CLVKGYFPEPVTVTWNSGSLSSGVHTFPAVLQSDLYTLSSSVTVPSSTWPSETVTCNVAHPASSTKVDKK-

	SP-VL-CL<br><br>MVSAIVLYVLLAAAAHSAFAMNIVLTQSPASLAVSLGQRATISCRASESLDSYGNSFMHWYQQKPGQPPKLLIYLASNLESGV<br>PARFSGSGSRTDFTLTIDPVEADDAATYYCQQNKEDLLTFGAGTKLELKRADAAPTVSIFPPSSEQLTSGGASVVCFLNNFY<br>PKDINVKWKIDGSERQNGVLNSWTDQDSKDSTYSMSSTLTLTKDEYERHNSYTCEATHKTSTSPIVKSFNRNECSN-
4B2<br>Fab	SP-VH-CH<br><br>MVSAIVLYVLLAAAAHSAFAMEVQLQQSGAELVRSGASVKLSCTGSGFNIKDYYMHWVKQRPEQGLEWIGWIDPENGDTE<br>YAPKFQGKATMTADTSSNTAYLHLSSLSSEDTAVYYCKGDYRYDDGGNYWGQGTSVTVSSAKTTAPSVYPLAPVCGDTTG<br>SSVTLGCLVKGYFPEPVTLTWNSGSLSSGVHTFPAVLQSDLYTLSSSVTVTSSTWPSQSITCNVAHPASSTKVDKKIEPRGP<br>TIKPC<br><br>SP-VL-CL<br><br>MVSAIVLYVLLAAAAHSAFAMDIQMTQTTSSLSASLGDRVTISCRASQDISNYLNWFQQKPDGTVKLLIYYTSRLHSGVPSRF<br>SGSGSGTDYSLTINNLEQEDIATYFCQQGDSLPWTFGGGTKLEIKRADAAPTVSIFPPSSEQLTSGGASVVCFLNNFYPKDIN<br>VKWKIDGSERQNGVLNSWTDQDSKDSTYSMSSTLTLTKDEYERHNSYTCEATHKTSTSPIVKSFNRNEC-
9B5<br>Fab	SP-VH-CH<br><br>MVSAIVLYVLLAAAAHSAFAMEVQLQQSGAELVRSGASVKLSCTTSGFNIKDYYMHWVKQRPEQGLEWIGWIDPENGDAEY<br>APKFQGKATMTADTSSNTAYLQLSSLTSEDTAVYYCNSGVAYWGQGTLVTVSAAKTTAPSVYPLAPVCGDTTGSSVTLGCL<br>VKGYFPEPVTLTWNSGSLSSGVHTFPAVLQSDLYTLSSSVTVTSSTWPSQSITCNVAHPASSTKVDKKIE-<br><br>SP-VL-CL<br><br>MVSAIVLYVLLAAAAHSAFAMDVVLTQTPLTLSVTIGQPASISCKSSQSLLDSAGKTYLNWLLQRPGQSPKRLIYLVSKLDSGV<br>PDRFTGSGSGTDFTLKISRVEAEDLGIYYCWQGTHFPRTFGGGTKLEIKRADAAPTVSIFPPSSEQLTSGGASVVCFLNNFY<br>PKDINVKWKIDGSERQNGVLNSWTDQDSKDSTYSMSSTLTLTKDEYERHNSYTCEATHKTSTSPIVKSFNRNEC-
2H1<br>Fab	SP-VH-CH<br><br>MGWSSIILFLVATATGVHSQVQLQQPGADLVRPGASVKLSCKASGYSFTSYWMTWVKQRPGQGLEWIGMIHPSDSETRLN<br>QKFKDKATLTVDKFSSTAYMQLSSPTSEDSALYYCARGDYYGTSFGGYFDVWGAGTTVTVSSAKTTAPSVYPLAPVCGDTT

	GSSVTLGCLVKGYFPEPVTLTWNSGSLSSGVHTFPAVLQSDLYTLSSSVTVTSSTWPSQSITCNVAHPASSTKVDKKIEPRG<br>PTIKPCPPCKCPAPNLLGGPSVFIFPPKIKDVLMISLSPIVTCVVVDVSEDDPDVQISWFVNNVEVHTAQTQTHREDYNSTLRV<br>VSALPIQHQDWMSGKEFKCKVNNKDLPAPIERTISKPKGSVRAPQVYVLPPPEEEMTKKQVTLTCMVTDFMPEDIYVEWTN<br>NGKTELNYKNTEPVLDSDGSYFMYSKLRVEKKNWVERNSYSCSVVHEGLHNHHTTKSFSRTPGK-<br><br>SP-VL-CL<br><br>MDSQAQVLILLLLWVSGTCGDIVMSQSPSSLAVSAGEKVTMSCKSSQSLLNSRTRKSYLAWYQQKPGQSPRLLIYWASTRY<br>SGVPDRFTGSGSGTDFTLTISSVQAEDLAVYYCKQSYTLRTFGGGTKLEIKRADAAPTVSIFPPSSEQLTSGGASVVCFLNNF<br>YPKDINVKWKIDGSERQNGVLNSWTDQDSKDSTYSMSSTLTLTKDEYERHNSYTCEATHKTSTSPIVKSFNRNEC
